# Mobile air monitoring to identify volatile organic compound distributions and potential hazard during the remediation of the East Palestine, Ohio train derailment

**DOI:** 10.1007/s10661-025-14038-x

**Published:** 2025-04-26

**Authors:** Mariana Saitas, Toriq Mustapha, Eva Vitucci, Oladayo Oladeji, Han-Hsuan D. Tsai, Carolyn Cannon, Ivan Rusyn, Albert A. Presto, Weihsueh A. Chiu, Natalie M. Johnson

**Affiliations:** 1 Department of Environmental and Occupational Health, Interdisciplinary Faculty of Toxicology, Texas A&M University, College Station, TX 77843 USA; 2 Department of Mechanical Engineering, Carnegie Mellon University, Pittsburgh, PA 15213 USA; 3 Department of Microbial Pathogenesis and Immunology, Texas A&M University, College Station, TX 77843 USA; 4 Department of Veterinary Physiology and Pharmacology, Interdisciplinary Faculty of Toxicology, Texas A&M University, College Station, TX 77843 USA

**Keywords:** Air toxics, Disaster response, Environmental emergency, Chemical

## Abstract

On February 3, 2023, a train carrying hazardous chemicals derailed in East Palestine, Ohio. Initial mobile air monitoring in mid-February 2023 revealed unique spatiotemporal patterns for a range of volatile organic compounds (VOCs). To determine long-term impacts on air quality, two follow up mobile air monitoring campaigns were carried out in March and April 2023 on public roadways using a proton transfer reaction time-of-flight mass spectrometer. This study aimed to characterize VOC distributions, identify hotspots, and characterize potential hazards. Concentrations of targeted VOCs benzene, toluene, and xylenes were lower overall in East Palestine in April compared to March. Overall, more compounds (*n* = 48) were identified using non-targeted analysis (NTA) in East Palestine in March compared with April (*n* = 30). Several of these VOCs were uniquely identified during March sampling, and among the commonly detected compounds, higher average concentrations were observed in East Palestine compared background levels. Spatial analysis revealed similar patterns of hotspots for benzene and additional VOCs identified from NTA, including C_3_H_4_O, C_4_H_8_O, and C_6_H_12_O. Using the EPA Hazard Comparison Dashboard, 49% and 40% of the identified VOCs were classified as “very high” or “high” hazards for eye and skin irritation, respectively. Notably, eye and skin irritation were among the common symptoms reported by the residents near the disaster site. Additionally, 29%, 18%, and 14% of the identified VOCs were classified as “very high” or “high” hazards for genotoxicity/mutagenicity, acute inhalation toxicity, and acute dermal toxicity, respectively. Collectively, these findings provide exposure data supportive of the reported health concerns and demonstrate the application of mobile monitoring coupled with NTA as a novel approach for rapid identification and mapping of potentially hazardous chemicals following an environmental disaster.

**Supplementary Information:**

The online version contains supplementary material available at 10.1007/s10661-025-14038-x.

## Introduction

In East Palestine, Ohio, a train containing hazardous chemicals derailed on February 3, 2023. Among very diverse cargo, the train contained tanks of vinyl chloride, diethylene glycol, isobutylene, butyl acrylates, and benzene and other chemicals (USEPA, 2023). The derailed cars were accompanied by fires strong enough to further damage the derailed cars (Coelho et al., 2024). In response to the chemical spill and derailment fires, a controlled burn of vinyl chloride was performed on February 6 along with an evacuation order for residents within a one-mile of the derailment and a shelter in place order for those in a one- to two-mile area surrounding the village of East Palestine (Coelho et al., 2024). After the orders were lifted on February 8, the US Environmental Protection Agency (USEPA) began initial stationary air monitoring for volatile organic compounds (VOCs) at two sampling sites (USEPA, 2023). Shortly after, our team carried out a mobile air monitoring campaign using a proton transfer reaction time-of-flight mass spectrometer (PTR-ToF–MS) on February 20–21. Importantly, initial mobile monitoring showed that average concentrations of benzene, toluene, xylenes, and vinyl chloride were below minimal risk concentrations for intermediate and chronic exposures, similar to EPA stationary monitoring data (Oladeji et al., 2023). Concentrations of acrolein, a known respiratory irritant, were relatively high in East Palestine compared with local background. Additional non-targeted analyses (NTA), based on analyte kinetics, identified several compounds at above local background concentrations with similar spatial distribution as acrolein, including CH_3_NO (identified as formamide); C_2_H_4_O_2_, C_3_H_6_O_2_, C_3_H_6_O_3_, C_11_H_20_O_2_ (identified as ethyl hexyl acylate); and C_17_H_12_O_2_ (identified as butyl acylate). Several other compounds were detected, however, with no clear spatial patterns, including C_3_H_6_O, C_4_H_8_O, C_5_H_8_, and C_6_H_10_.

When train derailment cleanup efforts began, including soil removal and stream aeration, redistribution of VOCs into the air became a concern. Many residents continued to report headaches, nausea, runny noses, burning noses and/or throats, cough or difficulty breathing, eye irritation, and rashes from potential outdoor and/or indoor exposure (National Academies and of Sciences, E & Medicine, 2024). Several members of the Centers for Disease Control and Prevention (CDC) team studying the effects of the train derailment in East Palestine, Ohio, also became briefly ill in late March, reporting symptoms similar to residents, including sore throat, headache, coughing, and nausea (National Academies and of Sciences, E & Medicine, 2024).

Given the potential for mobile air monitoring to provide exposure data following environmental disaster events, we carried out two follow up mobile air monitoring surveys on March 16 and April 12, 2023. Around these sampling campaigns, there were two key events that occurred. On March 14, a culvert for Sulphur Run was high-pressure washed (Coelho et al., 2024). On April 11, the EPA completed air sparging on the two streams that ran along the train tracks, Leslie Run and Sulphur Run, as a part of remediation efforts (Coelho et al., 2024). This study aimed to evaluate changes in ambient air VOC distributions in the resolution of the derailment during the cleanup phase, identify hotspots, and characterize potential hazards.

## Materials and methods

### Instrumentation

The mobile air monitoring for March and April 2023 was carried out with the Texas A&M University Mobile Responding to Air Pollution in Disasters (mRAPiD) van. The van is equipped with a sample inlet located on a boom on the roof. The distance between the sample inlet to the roof of the van when the boom is fully extended is 1.2 m. The sample inlet consists of 0.5″ Teflon tubing, and the ambient air is brought into the van via a mechanical pump. The Teflon tubing is connected to 1/16″ PEEK tubing, which pulls the ambient air from the tubing line into the IONICON Analytik (Innsbruck, Austria) proton transfer reaction time-of-flight mass spectrometer (PTR-ToF) 4000 at a FC-Inlet rate of 200 standard cubic centimeters per minute (sccm). The PTR-ToF–MS is a highly sensitive instrument and can measure VOCs as low as one part per trillion (ppt). For the sampling trips in March and April, hydronium (H_3_O^+^) was used as the reagent ion. In addition, the mRAPiD van is equipped with a Magellan MX- 500 weather system. The Magellan MX- 500 is located on the roof of the sampling van and collects GPS, temperature, humidity, wind speed, and direction data every second. When the GPS function is selected, the Magellan corrects for the wind speed and direction data.

Note, the PEEK tubing is run through a heated inlet that maintains constant temperature, minimizing the effect of varying ambient air temperature, as well as ambient humidity on PTR-ToF–MS operation. High levels of humidity can still interfere with PTR-ToF–MS operation; however, humidity levels during these sampling campaigns were average (described in the “Results” section).

#### Monitoring sites

**Fig. 1:**
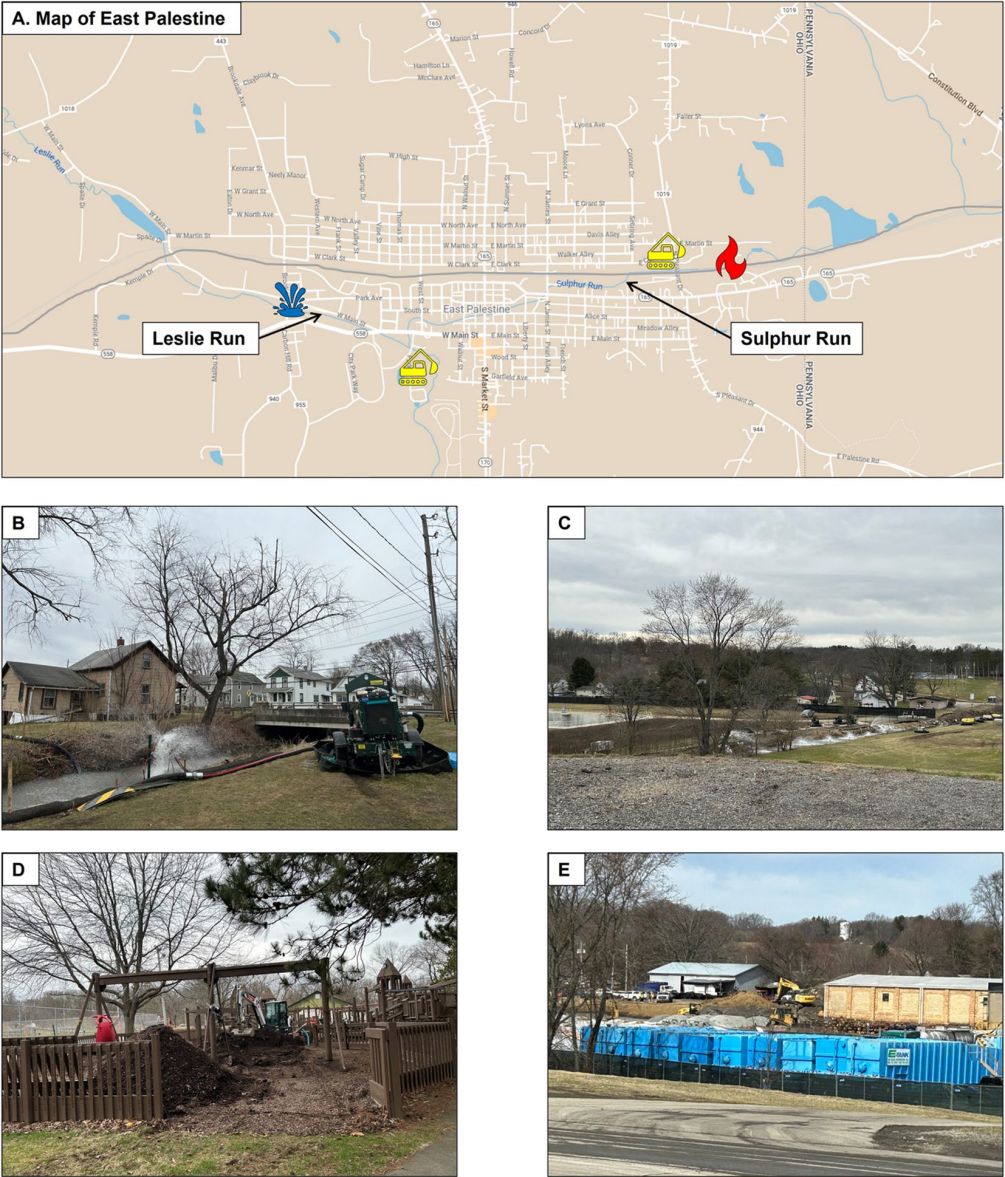
The USEPA issued an administrative order requiring Norfolk Southern to remove spilled substances and impacted soil and water in response to the train derailment and chemical spill (location shown in **A** by the red flame icon. The yellow excavator icons represent soil excavation; the blue water icons represent stream aeration. Photos taken by our study team on March 16, 2023, highlight stream aeration, in proximity to public roadways (**B**, **C**) and examples of soil removal at East Palestine Park (**D**) and near the derailment site (**E**)

Mobile monitoring was conducted in East Palestine, Ohio and Ohioville, Pennsylvania. Ohioville is a small town located ~ 16 miles south of East Palestine and was selected as the background based on similar environment and population. Monitoring with the mRAPiD van was conducted on publicly accessible roads at approximately 5 to 30 miles per hour (mph) based off similar speeds from other mobile sampling campaigns (Apte et al., 2017; Chambliss et al., 2021; Shah et al., 2018). A map of East Palestine is shown in Fig. 1 alongside photos taken from publicly accessible roadways during the March 16, 2023, sampling trip when the active phase of the contaminated soil removal and stream aeration (to off gas any volatile and semi-volatile chemicals that spilled into local waterways) was ongoing. A second sampling trip was conducted April 12, 2023, when stream aeration appeared inactive. The duration for background sampling was approximately 30 min at the start and end of each day. On March 16, mobile sampling in East Palestine occurred starting at 2:37 P.M. As we excluded the first 10 min to allow for equilibration of the PTR-ToF–MS detections, monitoring occurred from 2:47 P.M. to 5:50 P.M. On April 12, mobile sampling occurred from 9:14 A.M. (9:24 A.M. with the first 10 min excluded) to 5:08 P.M. The time stamps were recorded in UTC (coordinated universal time) and converted to CDT (central daylight time) using Microsoft Excel LTSC MSO (16.0.14332.20546) 64-bit to merge with the VOC data recorded in CDT.

### Data collection

VOC data collected from March and April 2023 were analyzed using the IONICON PTR-MS Viewer analysis software 3.4.4 following a workflow outlined in Figure S1. The VOC compounds used for targeted and non-targeted analysis had a threshold signal-to-noise ratio of 5 or above. For the April East Palestine data, the VOC and GPS data files were segmented and subsequently merged using RStudio version 4.2.3. We followed methods for targeted and non-targeted analyses previously described by Oladeji et al. (2023) and Vitucci et al. (2024) using the settings described in Table S1 and performed quality checks using standard gases (Table S2). For the targeted analysis, benzene, toluene, and xylene concentrations were multiplied by their corresponding calibration factor generated from a multipoint calibration curve (Figures S2− 4; Table S3). For the non-targeted analysis, peaks were identified using the IONICON compound database within the PTR-MS Viewer software. Compounds identified with at least 90% correctness were included in subsequent analyses. The default reaction rate constant of 2 × 10^− 9^ cm^3^ s^− 1^ was used to estimate VOC concentrations in ppbv. Note, the compound C_5_H_9_, commonly referred to as 1,3-butadiene, was excluded from analyses due to the poor resolution of this compound from background humidity when using H_3_O^+^ as the PTR-MS ion source. The selected peaks with at least 90% correctness were assigned a molecular formula. These filters resulted in a curated list of 64 molecular formulas. Using the IONICON database, we noted the possible compounds for each molecular formula. Some molecular formulas have one identity such as formic acid (CH_2_O_2_) whereas others such as C_5_H_10_O have eight possible compounds as multiple chemicals can be associated with the same molecular formula. All potential chemicals were then cross-referenced with EPA’s CompTox Dashboard and PubChem to further validate their identity (Table S5). Those that were not found in the EPA CompTox Dashboard or PubChem were denoted with an asterisk and excluded from further analysis.

### Data analysis

Wind direction was visualized and analyzed using non-parametric statistics in Oriana v.2. The Rayleigh test determined average wind direction, and the Watson-Wheeler test was performed to determine whether wind direction differed among sampling days in East Palestine. Statistical analysis for temperature, relative humidity, and corrected wind speed was conducted in GraphPad Prism (version 9.3.1) using a two-tailed, unpaired *t* test.

To identify relationships between the VOCs emitted and weather parameters across surveys, a correlation matrix was computed using the estimated average concentration of the curated VOCs in RStudio (version 4.2.3) (Figures S5 - 8 and S16). Spearman’s rank correlation analysis was used to identify significant relationships (*p* < 0.05). The 64 VOCs of interest were further analyzed using a ratio approach to compare relative abundance between: (1) East Palestine/Ohioville (EP/OH) and (2) March/April (M/A). The ratios of the average, median, and 95 th percentile were calculated for both EP/OH and M/A using a ratio analysis detailed in the Supplemental Methods section (Tables S6 - 7). For spatial mapping of the emitted VOCs, RStudio (version 4.2.3) was used to merge and visualize the VOC data with the Magellan MX- 500 data to produce maps showing concentration intensity along the sampling routes. The R packages used are tidyverse, ggmap, readxl, lubridate, ggpubr, googleway, corrplot, and ggthemes. Last, we used the EPA Hazard Comparison Dashboard (Williams et al., 2017) to evaluate the potential health hazard of identified VOCs. We limited this analysis to VOCs uniquely identified in East Palestine or those with average concentrations higher than the local background (Ohioville) during either the March or April sampling trip.

## Results

### Mobile air monitoring in East Palestine in March and April 2023

Following the train derailment, not only were a mixture of chemicals spilled, but there were fires among the wreckage. Soon after, a controlled burn was performed in early February 2023. After all of these events, the EPA issued a Superfund unilateral administrative order requiring Norfolk Southern to remove spilled substances and impacted soils from the derailment site (shown in Fig. 1A). Surface water bodies Sulphur Run, Leslie Run, and downstream creeks were included for remediation. In March 2023, remediation efforts were active, including soil removal and stream aeration. Photos taken by our study team on March 16, 2023, highlight soil removal near the derailment site and at East Palestine Park (Fig. 1B, C). Examples of stream aeration are also shown from publicly accessible roads (Fig. 1D, E) nearby Sulphur Run and Leslie Run, respectively. Remediation efforts continued into the spring; however, during our second sampling visit on April 12 th, 2023, stream aeration and soil excavation was not active.

### Concentrations of target chemicals BTX

**Fig. 2:**
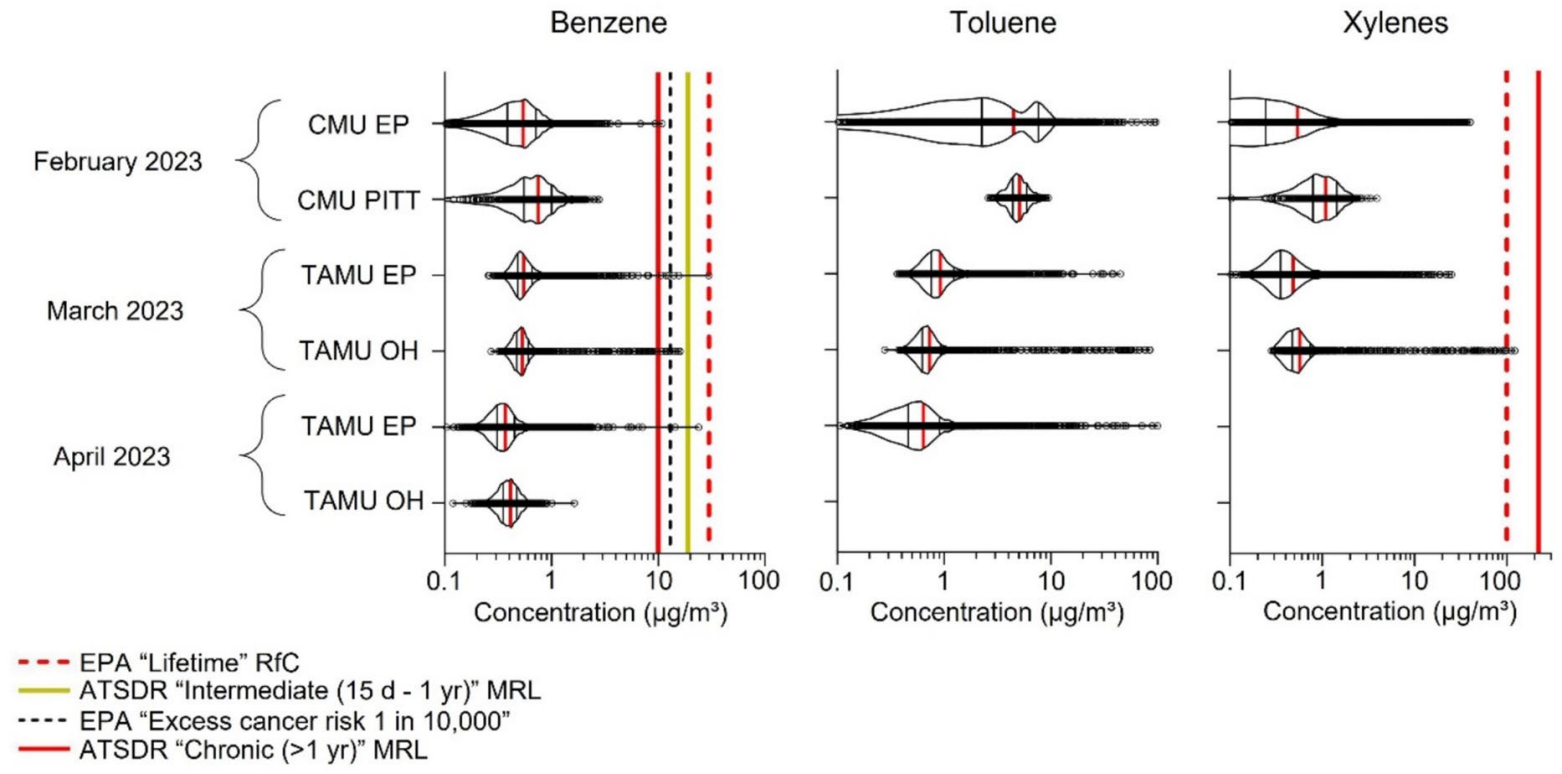
Concentrations of benzene, toluene, and xylenes in February collected using the Carnegie Mellon University (CMU) mobile air quality laboratory in East Palestine (EP), Pittsburgh (PITT), and from follow-up monitoring in March and April using the Texas A&M University (TAMU) mobile Responding to Air Pollution in Disasters (mRAPiD) van in EP and Ohioville (OH). The violin plots and reference values are modeled after Oladeji et al. (2023) showing the concentrations of select compounds during remediation

During sampling on March 16 and April 12, 2023, we carried out targeted analysis for benzene, toluene, and mixed xylenes (BTX, Fig. 2), since we could quantify based on calibration curves (Figures S2− 4). All the BTX median concentrations were below reference values set by the EPA and ATSDR (Table S4). Toluene is not classified as carcinogenic to humans; thus, no excess cancer risk value could be derived, and there is no ATSDR intermediate (15 days–1 year) minimal risk level (MRL). Reference values for the EPA “Lifetime” reference concentration (RfC) and the ATSDR “Chronic (> 1 year)” MRL are not present in the toluene violin plot due to the values being greater than 100 μg/m^3^.

### Non-targeted analysis of mobile air sampling data

**Fig. 3:**
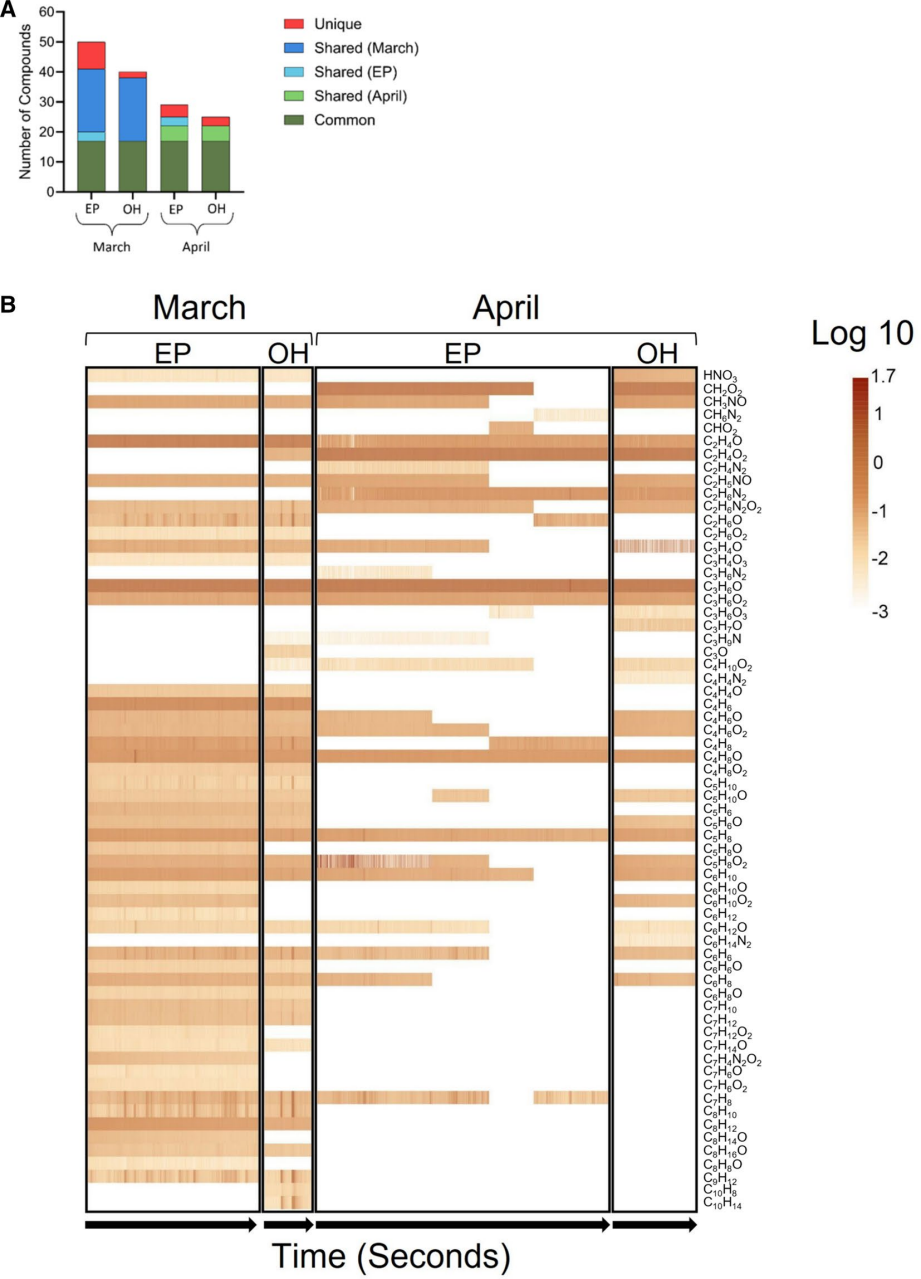
**A** Number of VOCs detected from each sampling location (East Palestine, EP or Ohioville, OH) in March and April. “Common” VOCs (dark green) are compounds detected at all sampling locations and both months. “Shared” VOCs are compounds detected at multiple, but not all, sampling locations and months. “Unique” VOCs (red) are compounds detected at only one sampling location in one specific month. **B** Overall raw concentration heatmap for each location and month. The raw concentrations were transformed using log 10 and are shown over time in seconds. The compounds are organized by carbon number from top to bottom

In addition to benzene, toluene, and xylene, NTA of the March and April 2023 mobile sampling data identified 64 additional molecular compounds. More compounds were identified from NTA in March compared to April (Fig. 3A). In March, 48 compounds were identified in East Palestine, and 44 were identified in Ohioville. In April, 30 compounds were identified in East Palestine, and 28 were identified in Ohioville. “Common” VOCs were defined as compounds detected at all sampling locations and both time points. “Shared” VOCs were defined as compounds detected at multiple, but not all, sampling locations and time points. Last, “unique” VOCs were defined compounds detected at only one sampling location in a specific month. Figure S9 also shows a Venn diagram of the number of unique and overlapping VOC compounds identified across all four sampling runs in March and April. In East Palestine, there were nine unique VOCs (C_5_H_8_O, C_6_H_12_, C_6_H_10_O, C_7_H_6_O_2_, C_7_H_12_O_2_, C_7_H_6_O, C_7_H_4_N_2_O_2_, C_8_H_14_O, and C_8_H_8_O) identified in March and four unique VOCs (C_2_H_4_N_2_, C_3_H_6_N_2_, CHO_2_, and CH_6_N_2_) identified in April. A heatmap of raw concentrations of each identified compound (Fig. 3B) displays the log-transformed concentrations for identified VOCs detected in East Palestine and Ohioville in March and April.

**Fig. 4:**
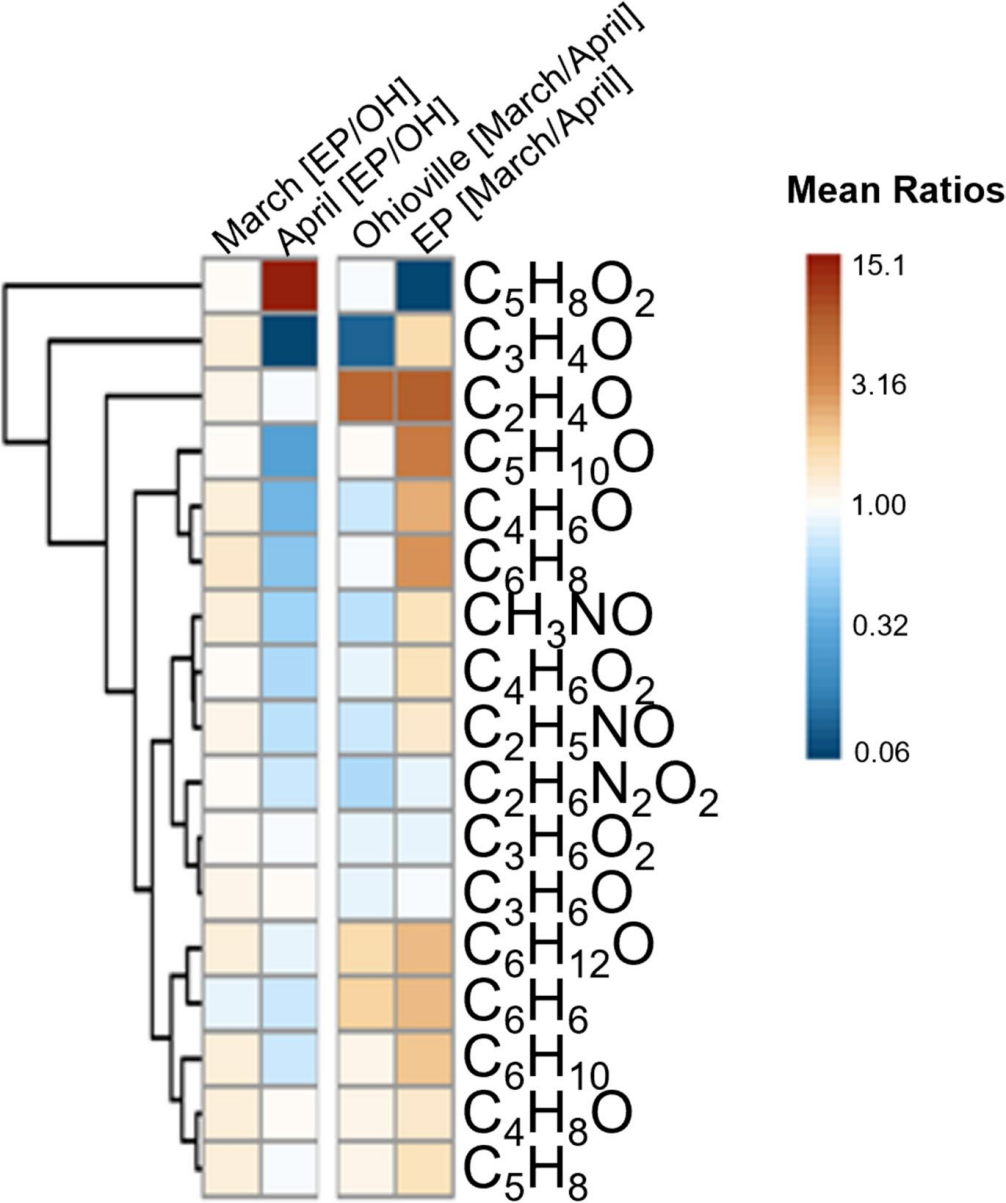
A heatmap showing the mean concentration ratios of 17 selected the common VOCs between East Palestine/Ohioville (EP/OH) and March/April (M/A). Ratios were computed for analytes determined via non-targeted analysis showing mean concentrations in East Palestine divided by Ohioville (EP/OH) in March and April, left panel, and relative concentrations in March divided by April (M/A) for Ohioville and East Palestine, right panel. The compounds (rows) are clustered using Euclidean distance and complete linkage

Additionally, we selected the 17 common VOCs for additional comparisons. These included compounds that were detected by NTA in both East Palestine and Ohioville in both months. These 17 VOCs were analyzed using a ratio approach to compare the relative average concentrations between (1) East Palestine/Ohioville (EP/OH) and (2) March/April (M/A) (Fig. 4). Most compounds showed a similar ratio (~ 1) in March, indicating relatively similar concentrations. In April, several of the compounds showed ratios < 1, indicating slightly higher average concentrations in Ohioville. Two exceptions were C_5_H_8_O_2_ and C_3_H_4_O. C_5_H_8_O_2_ was much higher in East Palestine in April (ratio ~ 15), whereas C_3_H_4_O was much lower in East Palestine in April (ratio ~ 0.06). When comparing monthly variation within each location, in Ohioville, C_3_H_4_O was much higher in April (indicated by a low ratio). Conversely, relative mean concentrations of C_2_H_4_O in Ohioville were higher in March. This compound was also relatively higher in East Palestine in March, compared to April. Overall, most compounds had relatively higher average concentrations in March versus April in East Palestine. One notable exception was C_5_H_8_O_2_, which had a ratio < 1 (i.e., higher relative average concentration in April).

### Spatial patterns for VOCs

**Fig. 5:**
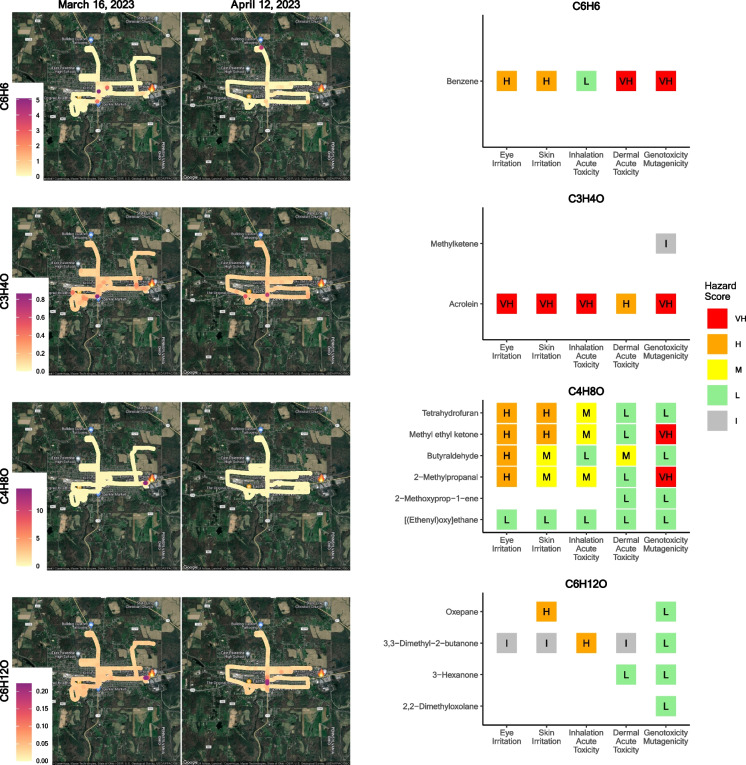
Select compound concentration maps with similar hotspots in East Palestine during March and April showing benzene (C_6_H_6_), C_3_H_4_O, C_4_H_8_O, and C_6_H_12_O, left panel. All potential names of the chemical formulas were entered into the EPA Hazard Comparison Dashboard to indicate potential hazard for eye irritation, skin irritation, inhalation acute toxicity, dermal acute toxicity, and genotoxicity/mutagenicity. The hazard scores indicate very high (VH), high (H), medium (M), low (L), and inconclusive (I) risks

Spatial analysis showed similar hotspots for four compounds along roadways in East Palestine (Fig. 5, left panel), including C_6_H_6_ (benzene), C_3_H_4_O (potentially representing methyl ketene or acrolein), C_4_H_8_O (potentially representing tetrahydrofuran; methyl ethyl ketone; butyraldehyde; 2-methylpropanal; 2-methoxyprop- 1-ene; or [(ethenyl)oxy]ethane), and C_6_H_12_O (potentially representing oxepane; 3,3-dimethyl- 2-butanone; 3-hexanone; or 2,2-dimethyloxolane). Comparisons of these four compounds between East Palestine and Ohioville (local background) are shown in Figure S10. In the February 2023 survey, there were similar spatial patterns between C_3_H_4_O and four compounds (CH_3_NO, C_2_H_4_O_2_, C_3_H_6_O_3_, and C_3_H_6_O). To determine if there were similar spatial patterns of these five compounds in the March and April 2023 campaigns, comparisons are shown in Figures S10 - 11. Only three of the five compounds, including C_3_H_4_O, formamide (CH_3_NO), and C_3_H_6_O, were detected in the March survey. In March, localized hotspots for CH_3_NO and C_3_H_6_O were detected around roadways nearby East Palestine City Park and on roadways towards the center of the town. For C_3_H_4_O, a localized hot spot was detected on roadways towards the center of the town. In comparison, in the April survey, all five compounds were detected. In April, the localized hot spots for CH_3_NO, C_2_H_4_O_2_, and C_3_H_6_O were detected on roadways nearby the East Palestine City Park. For C_3_H_6_O_3_, a hot spot was detected on a roadway near Leslie run. For C_3_H_4_O, hot spots were detected on roadways in the vicinity of Leslie Run, East Palestine City Park and towards the center of town.

#### Meteorological data

Temperature, relative humidity, and wind speed and direction data were collected in real-time (Supplemental Tables S11 and S12). Relative humidity (RH) and temperature measurements varied between March and April (Supplemental Figures S14 and S15). RH and temperature were significantly higher in April. Additionally, wind speed and direction varied across months, reflecting unique sampling conditions. Correlation analysis (Supplemental Figure S16) showed temperature and relative humidity were significantly, weakly correlated with the detection of VOCs in March. In April, the majority of VOCs showed a strong, significantly positive correlation with humidity and a strong, significantly negative correlation with ambient temperature.

### Hazard characterization

**Fig. 6:**
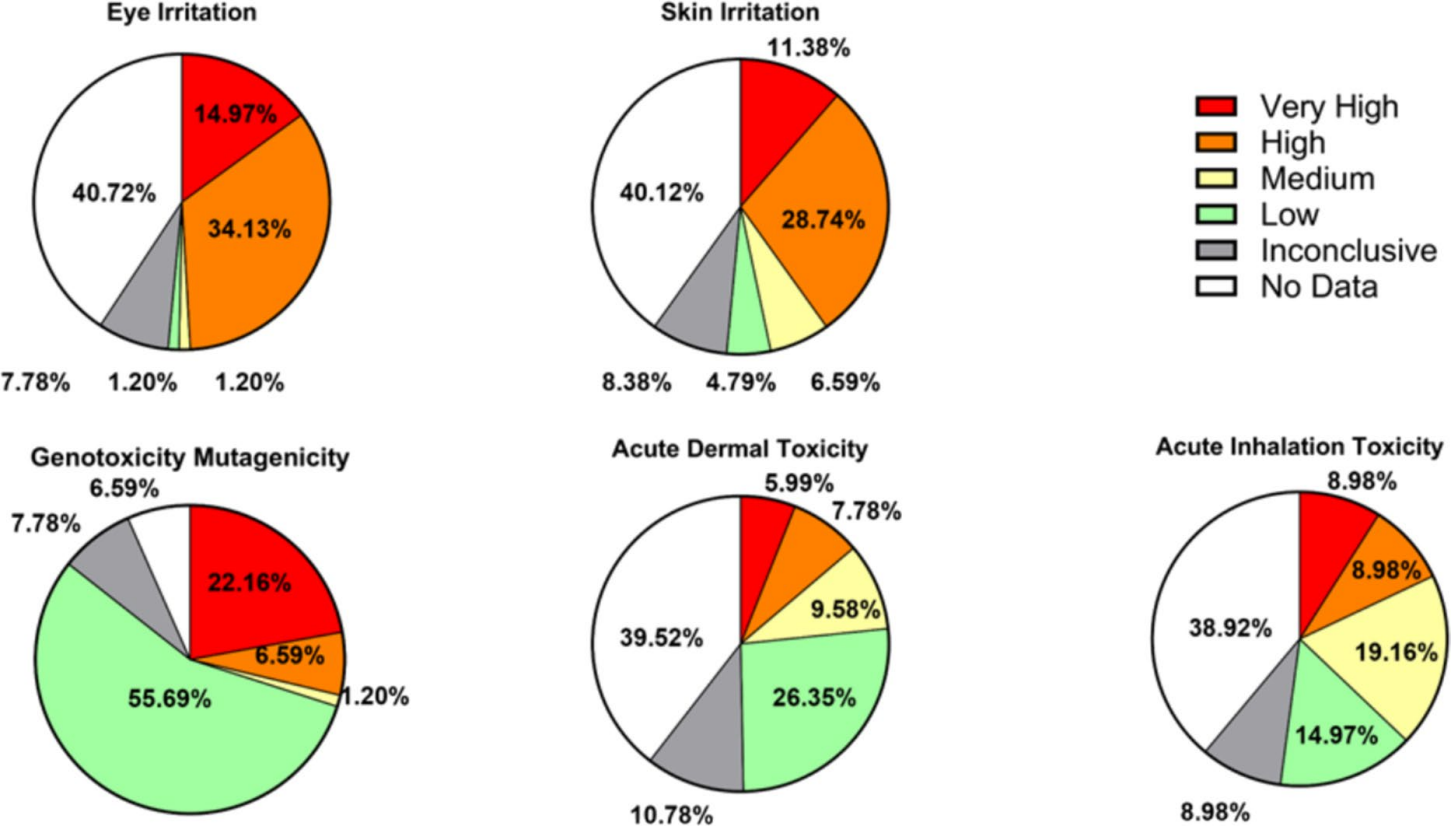
VOCs uniquely identified in East Palestine in March and April sampling trip were all entered into the EPA Hazard Comparison Dashboard. Pie charts show the relative hazards (or lack of data) across each category

We used the EPA Hazard Comparison Dashboard (Williams et al., 2017) to evaluate the potential health hazard of identified VOCs found to be elevated East Palestine. For the four compounds with similar spatial patterns (Fig. 5), hazard is shown in the right panel for all potential known chemical names. For C_6_H_6_ (benzene), we also confirmed this chemical through targeted analysis (Fig. 2). Benzene has a high hazard for eye and skin irritation. It has a low risk for acute inhalation toxicity and a very high risk for acute dermal toxicity and genotoxicity mutagenicity. For C_3_H_4_O (acrolein or possibly methyl ketene), all hazard categories are very high, and acute dermal irritation is high risk. For C_4_H_8_O and C_6_H_12_O, there could be many potential chemical names based on the chemical formula. To show all potential hazards present, hazards from these possible chemicals are shown to range from low to very high. For a number of chemicals, the risk is inconclusive or absent (incomplete). Overall, for C_4_H_8_O, most risk is associated with eye or skin irritation. This was also the case for the majority of chemicals identified through NTA (Fig. 6).

Figure 6 shows the hazard information for VOCs uniquely identified in East Palestine or with average concentrations higher than the local background (Ohioville) during either March or April (Supplemental tables S8 - 10). Pie charts show the relative hazards (or lack of data) across each category, including eye irritation, skin irritation, genotoxicity mutagenicity, acute dermal toxicity, and acute inhalation toxicity. Approximately 49% and 40% of the predicted VOCs were classified as either “very high” or “high” hazards for eye and skin irritation, respectively. Approximately 29% of the predicted VOCs were associated with either a very high or high genotoxic hazard. Acute dermal and inhalation toxicity had the fewest number of predicted VOCs classified as either very high or high, with ~ 14% and 17%, respectively. Notably, while most of the identified VOCs had data to support their classification as a health hazard for genotoxicity mutagenicity, approximately 40% of the predicted VOCs had no data on eye irritation, skin irritation, acute dermal toxicity, or acute inhalation toxicity hazard.

## Discussion

VOCs are produced from a variety of natural and anthropogenic sources. Numerous VOCs are classified as hazardous air pollutants based on their potential for adverse human health effects (Zhou et al., 2023). The EPA is required to regulate emissions of hazardous air pollutants under the Clean Air Act, and common VOCs are routinely monitored through a network of stationary air monitors (USEPA, 2023). The emergence of mobile air monitoring over the past decade has been driven by multiple factors, including its use as a tool to address air quality data gaps (Wang et al., 2023). Emissions from environmental disaster events further highlight the potential for mobile air monitoring to facilitate agile air pollution mapping to compliment routine stationary air monitoring (Bhandari et al., 2022).

We initially conducted mobile air monitoring in East Palestine in the aftermath of the train derailment in mid-February 2023 (Oladeji et al., 2023). There were subsequent mobile air monitoring campaigns conducted by the EPA’s Trace Atmospheric Gas Analyzer (TAGA) bus and reportedly contracted by Norfolk Southern using PTR-MS (USEPA, 2023). However, the TAGA bus only measured for *n*-butyl acrylate and vinyl chloride, and PTR-MS data was not publicly available (USEPA, 2023). Based on initial findings (Oladeji et al., 2023), our team decided to sustain sampling efforts in March and April as cleanup efforts were underway. The follow-up mobile laboratory to East Palestine was conducted on March 16 and April 12, 2023, along public roadways throughout the town. First, we quantified concentrations of benzene, toluene, and xylene in ambient air and made comparisons to local background in Ohioville, Pennsylvania. In March, median benzene and toluene concentrations were slightly higher in East Palestine compared to Ohioville, whereas median xylene levels were slightly higher in Ohioville. Benzene has many common sources, including gasoline emissions and cigarette smoke. Toluene is also added to gasoline and used as a solvent, and xylene is similarly present in vehicle exhaust (Zhou et al., 2023). The comparable ranges of benzene, toluene, and xylene (BTX) in East Palestine and Ohioville highlight their common sources that are likely unrelated to the train derailment. This was not unexpected due to the different chemicals onboard train cars. Still, disaster events, including the cleanup phase may increase traffic-related air pollution, including BTX (Bhandari et al., 2022). Overall, median BTX concentrations decreased from March to April sampling periods in both locations. In April, median benzene concentrations were similar in East Palestine and Ohioville. In April, toluene was still detected in East Palestine, but not in Ohioville, and xylene was not detected in either location. The differences between March and April could be due to multiple factors, including seasonal variation or different metrological conditions.

Time of day and different meteorological conditions can also contribute to unique VOC signatures. Our sampling was conducted at around the same time of day for each trip in March and April; however, the temperature, relative humidity, wind speed, and wind direction were significantly different between each sampling day. On March 16, the maximum and minimum temperatures detected in East Palestine were 57.9 °F and 56.2 °F, whereas in Ohioville, the maximum and minimum temperatures were 56.9 °F and 54.2 °F. On April 12, the maximum and minimum temperatures detected in East Palestine were 80.9 °F and 75.4 °F, whereas in Ohioville, the maximum and minimum temperatures were 71.6 °F and 68.1 °F. Average relative humidity in East Palestine was 20% and 30% in March and April, respectively. A correlation analysis between VOC detection, temperature, and relative humidity suggested these weather parameters were significantly, weakly correlated with the detection of varying of VOCs in March. Interestingly, this relationship was much larger in April, with the majority of VOCs having a strong, significantly positive correlation with humidity and a strong, significantly negative correlation with ambient temperature. Together, these findings suggest these weather parameters were more associated with the VOC detection in April than March and may contribute to the differing results between the sampling campaigns. Significant, yet slight changes in average wind speed (5.3 mph and 7.0 mph in March and April, respectively) and wind direction (233.1° and 282.5° in March and April, respectively) in East Palestine were also observed, which may further contribute to the observed differences.

Notably, all median BTX concentrations were below different types of health-protective concentrations of exposure. For benzene, we compared measured concentrations to reference values including the EPA lifetime RfC, EPA excess cancer risk 1 in 10,000, ATSDR chronic (> 1 year) MRL, and ATSDR intermediate (15 days–1 year) MRL. All median benzene concentrations were below these concentrations. Some spikes detected in East Palestine in March and April and Ohioville in March did exceed health protective limits, but the interpretation of risk from a very acute spike (i.e., a few seconds) is currently unclear. Notably, mobile air monitoring is a rapidly developing technology without direct comparisons to regulatory limits (i.e., second-level resolution from mobile air monitoring versus daily/annual averages used by stationary monitors) (Wang et al., 2023). We acknowledge that making short-term comparisons to chronic health-protective concentrations of exposure is not a direct comparison; however, we sought to contextualize exposures based on past reports (Bhandari et al., 2022; Oladeji et al., 2023; Vitucci et al., 2024). The EPA 8-h acute exposure guideline level (AEGL) for benzene is 29,000 μg/m^3^, which is substantially higher than measured levels. AGELs are used when dealing with accidental releases of chemicals into air and are designed to protect susceptible populations, including the elderly and children. In addition, all median xylene concentrations were below the EPA lifetime RfC and the ATSDR chronic MRL. As toluene is not classified as carcinogenic to humans, there is no excess cancer risk value and no ATSDR intermediate MRL. Reference values for the EPA Lifetime RfC and the ATSDR chronic MRL are not present in the toluene violin plot due to the values being greater than 100 μg/m^3^.

A major strength of PTR-ToF–MS is its ability to identify potentially novel VOCs that may not be routinely monitored through non-targeted analysis. Non-targeted analysis is a broadly defined concept involving the characterization of the chemical composition of any given sample without the use of a priori knowledge regarding the chemical content (Place et al., 2021). Thus, while our team routinely calibrates based on a set of known chemical standards to quantify absolute concentrations of specific VOCs, PTR-ToF–MS technology can identify relative concentrations of a wide range of VOCs. The application of non-targeted analysis has been suggested as a useful tool in disaster response to aid in identifying emerging chemical contaminants (Phillips et al., 2022; Sloop et al., 2023). Furthermore, the presence of the detected chemicals and their respective relative concentrations could be influenced by the remediation activity from the EPA and other contractors due to commercial products that were used to address odors at the derailment site (Coelho et al., 2024). To our knowledge, this is one of the few applications of mobile air monitoring to apply PTR-ToF–MS in a disaster response to identify unknown VOCs.

Following our quality control parameters, we detected 48 compounds from non-targeted analysis in East Palestine in March and 30 compounds in April. A similar number of compounds were also detected in the local background (Ohioville) in March and April. In total from both surveys in March and April, 17 common compounds were identified. Interestingly, there were nine unique compounds detected in East Palestine in March and four unique compounds detected in April. None of these compounds were uniquely identified in East Palestine in February from prior sampling (Oladeji et al., 2023), which reflects the presence of distinct VOCs across all 3 months in the aftermath of the train derailment and chemical spill.

Some of the compounds identified had unique relative concentration changes between sampling months and locations. C_3_H_4_O, identified as acrolein or potentially methyl ketene based on the chemical formula, remained relatively higher in East Palestine compared to Ohioville. For C_3_H_4_O, hot spots were also detected on roadways in the vicinity of Leslie Run, East Palestine City Park, and on roadways towards the center of town. Acrolein has several common sources, including emissions from vehicle exhaust, fires, and tobacco. Several health hazards are well known, including skin and eye irritation, acute inhalation and dermal toxicity, and genotoxicity. Alternatively, little is known about potential health hazards of methyl ketene, and sources are not as common. Additionally, C_5_H_8_O_2_ was much higher in East Palestine in April. This formula can represent many potential chemicals. Overall, when mapping chemicals with similar hotspots in East Palestine, benzene (C_6_H_6_), C_3_H_4_O, C_4_H_8_O, and C_6_H_12_O showed similar spatial patterns. Prior work following Hurricane Florence revealed an association between higher concentrations of benzene and fine particulate matter near fueling stations, particularly immediately after the hurricane (Bhandari et al., 2022). Several fueling stations are present in East Palestine, however, patterns indicated hotspots near the derailment site that is located east of most fueling station locations. Increased excavation activity using heavy equipment present at the time near the derailment site could present a potential source of chemical exposures. C_4_H_8_O could be many chemicals, including butyraldehyde, a manufactured chemical, but it is also present in the environment from natural sources. It is also released to the air in vehicular emissions. Collectively, looking across all the potential chemical names for C_4_H_8_O, nearly all represent a high hazard for eye irritation. Likewise, when comparing all chemicals present or elevated in East Palestine, the highest parentage of very high risk (based on the EPA dashboard) was for eye irritation. This matches with the symptoms reported by residents. The next most frequent very high hazard was for skin irritation, which was also commonly reported by residents. Notably, many residents reported symptoms both outside and inside their homes (National Academies and of Sciences, E & Medicine, 2024). This study was not designed to evaluate indoor exposure to VOCs.

Our study has several limitations. As we used NTA, we were unable to definitively assign chemical names to all identified formulas. Likewise, we could not quantify absolute chemical concentrations for compounds for which we did not have standards. This leads to some level of uncertainty in the identity of the chemicals present at specific concentrations. In addition, although we demonstrate high spatial resolution, the temporal coverage was low, representing only 1 day in March and 1 day in April. Lastly, our sampling routes only covered roadways in East Palestine, and no sampling was done in driveways or in residents’ homes. The exposures identified are not fully representative of residential exposure.

## Conclusions

Concentrations of targeted VOCs benzene, toluene, and xylene were lower overall in East Palestine in April than in March. Spatial analysis revealed similar patterns of hotspots for benzene and additional chemical formulas identified from NTA, including C_3_H_4_O, C_4_H_8_O, and C_6_H_12_O. Collectively, our findings demonstrate the application of mobile monitoring coupled with NTA as a novel approach to identify chemical distributions following environmental disasters. This study highlights the presence of complex VOC mixtures in East Palestine during remediation and the need for additional toxicology data to inform hazard types.

## Supplementary Information

Below is the link to the electronic supplementary material.

Supplementary file1 (DOCX 3544 KB)

Supplementary file2 (XLSX 612 KB)

## Data Availability

Data is provided within the manuscript or supplementary information files.
