# Supplementary material for: Mobile air monitoring to identify volatile organic compound distributions and potential hazard during the remediation of the East Palestine, Ohio train derailment
Source: Environ Monit Assess. 2025 Apr 26;197(5):597. doi: 10.1007/s10661-025-14038-x (PMC12033195; doi:10.1007/s10661-025-14038-x)
Supplement: Supplementary file 1 — Supplementary file1 (DOCX 3544 KB) [file 10661_2025_14038_MOESM1_ESM.docx]

**Supplemental Information**

**Mobile Air Monitoring to Identify Volatile Organic Compound Distributions and Potential Hazard During the Remediation of the East Palestine, Ohio Train Derailment**

Mariana Saitas1, Toriq Mustapha1, Eva Vitucci1, Oladayo Oladeji2, Han-Hsuan D. Tsai4, Carolyn Cannon3, Ivan Rusyn4, Albert A. Presto2, Weihsueh A. Chiu4 and Natalie M. Johnson1*

1 Department of Environmental and Occupational Health, Interdisciplinary Faculty of Toxicology, Texas A&M University, College Station, Texas 77843, United States.

2 Department of Mechanical Engineering, Carnegie Mellon University, Pittsburgh, Pennsylvania 15213, United States.

3 Department of Microbial Pathogenesis and Immunology, Texas A&M University, College Station, Texas 77843, United States.

4 Department of Veterinary Physiology and Pharmacology, Interdisciplinary Faculty of Toxicology, Texas A&M University, College Station, Texas 77843, United States.

*Corresponding author: [nmjohnson@tamu.edu](mailto:nmjohnson@tamu.edu) (ORCID: 0000-0001-8584-9713)

**Supplemental Methods**

1. **Workflow**

Figure S1 outlines the workflow of the disaster response research in East Palestine, Ohio. These steps include data collection, analysis, and visualization.

**Figure S1.** Steps taken for March and April 2023 campaigns in East Palestine.

1. **Data analysis**

VOC data is analyzed using the PTR-MS Viewer software. After uploading the desired files, a mass calibration is performed for each file. The purpose of the mass calibration is to ensure that the masses are accurate by using a 3-point mass calibration (hydronium, acetone, and 1,3-diiodobenzene). The RMS Mass Error we look for should be less than .001. After the mass calibration, we use the peaktable and set the signal-to-noise ratio to 5 to ensure the compounds detected are truly in the environment. This ratio produces a list of identified VOCs. From there we select the peaks for compounds with a mass of 45 and above. After the peaks are selected, the percent correctness for each peak is analyzed and only the peaks with a 90% correctness are chosen. The higher the percent correctness, the more likely the compound is accurately identified. The final list of identified VOCs is then exported for further analysis for targeted and non-targeted.

1. **PTR-ToF-MS 4000**
   1. **Quality Check**

In total, we used two standard gases to perform quality checks for the PTR-Tof-MS. In the March 2023 campaign, we used a standard gas prepared by Scotty Analyzed Gases. The compounds in the standard gas are listed in Table S1 for quality check prior to each campaign. For the April 2023 campaign, a standard gas prepared by the RJ Lee Group was used. The compounds in the RJ Lee Group standard gas are listed in Table S2. The quality check of the PTR-ToF-MS with the standard gas ensures the readings of the PTR-ToF-MS in-field would be accurate.

**Table S1**. Compounds in standard gas for quality check during March 2023 campaign.

| Formula | Name | Mass | Concentration in standard (ppb) |
| --- | --- | --- | --- |
| C4H6 | 1,3-Butadiene | 54.09 | 100.000 |
| C3H4O | Acrolein | 56.06 | 100.000 |
| C8H10 | Mixed Xylene | 106.16 | 100.000 |
| C8H10 | Ethylbenzene | 106.16 | 100.000 |
| C6H6 | Benzene | 78.11 | 100.000 |
| C7H8 | Toluene | 92.14 | 100.000 |
| C8H8 | Styrene | 104.15 | 100.000 |

**Table S2.** Compounds in standard gas for quality check during April 2023 campaign.

| Formula | Name | Mass | Concentration in standard (ppb) |
| --- | --- | --- | --- |
| CH4O | Methanol | 32.026215 | 2126 |
| C2H3N | Acetonitrile | 41.026549 | 2316 |
| C3H6O | Acetone | 58.041865 | 2260 |
| C2H3Cl | Vinyl Chloride | 61.992328 | 2171 |
| C5H8 | Isoprene | 68.0626 | 2229 |
| C4H6O | Methyl Vinyl Ketone | 70.041865 | 2217 |
| C4H8O | 2- Butanone | 72.057515 | 2256 |
| C6H6 | Benzene | 78.04695 | 2239 |
| C7H8 | Toluene | 92.0626 | 2242 |
| C2H2Cl2 | 1,2-Dichloroethylene | 96.95671 | 2254 |
| C8H8 | Styrene | 104.0626 | 2271 |
| C8H10 | p-Xylene | 106.07825 | 2210 |
| C9H12 | 1,3,5-Trimethylbenzene | 120.0939 | 2221 |
| C2HCl3 | Trichloroethylene | 129.914383 | 2233 |
| C10H14 | 1,2,3,5 Tetramethylbenzene | 134.10955 | 2319 |
| C2Cl4 | Tetrachloroethylene | 165.872461 | 2193 |

1. **Targeted Analysis**
   1. **Compound Conversions and Calibration Factor**

The values produced from the PTR-ToF-MS 4000 are the raw concentrations (ppbv). To attain the true concentration of the compounds of interest (BTX compounds), the raw concentrations need to be multiplied by their respective calibration factors which are listed in Table S3. A multi-point calibration was performed to derive these calibration factors and can be seen in Figures S2-S4.

**Figure S2.** Multi-point calibration of benzene.

**Figure S3.** Multi-point calibration of toluene.

**Figure S4.** Multi-point calibration of xylenes.

**Table S3.** Calibration factors for the BTX compounds.

| Name | CASRN | Calibration Factor |
| --- | --- | --- |
| Benzene | 71-43-2 | 1.8305 |
| Toluene | 108-88-3 | 2.6907 |
| Xylenes | 1330-20-7 | 2.3359 |

After multiplying the raw concentrations of the BTX compounds by their respective calibration factors, the following equation is used to convert from ppbv to μg/m3.1

.

For the visualization of the targeted analysis data from March and April 2023, the format in addition to the reference level values for the BTX compounds were made to be consistent with the previous study done by Oladeji et al.2 In Table S4, the reference level type, value, and their source are listed.

**Table S4.** BTX compounds and their respective reference levels and values.

| Chemical Name | Reference Level Type | Value (μg/m3) | Source |
| --- | --- | --- | --- |
| Benzene (71-43-2) | Chronic RfC | 30 | IRIS |
| Benzene (71-43-2) | Chronic MRL | 10 | ATSDR |
| Benzene (71-43-2) | Intermediate MRL | 19 | ATSDR |
| Benzene (71-43-2) | 10-4 Cancer Risk | 13 | IRIS |
| Toluene (108-88-3) | Chronic RfC | 5000 | IRIS |
| Toluene (108-88-3) | Chronic MRL | 3800 | ATSDR |
| Xylenes (1330-20-7) | Chronic RfC | 100 | IRIS |
| Xylenes (1330-20-7) | Chronic MRL | 220 | ATSDR |
| Xylenes (1330-20-7) | Intermediate MRL | 2600 | ATSDR |

ATSDR: Agency for Toxic Substances and Disease Registry; IRIS: Integrated Risk Information System (U.S. EPA); MRL: Minimal Risk Level; RfC: Reference Concentration

1. **Non-Targeted Analysis Data Visualization**

An R-script was developed to analyze the non-targeted data and produce a series of visualizations. One of the visualizations the script produced were correlation plots. The correlation plots for East Palestine and Ohioville from the March and April 2023 campaigns are listed in Figures S5-S8.

- 1. **Correlation Plots**

**Figure S5.** East Palestine March 2023 correlation plot.

**
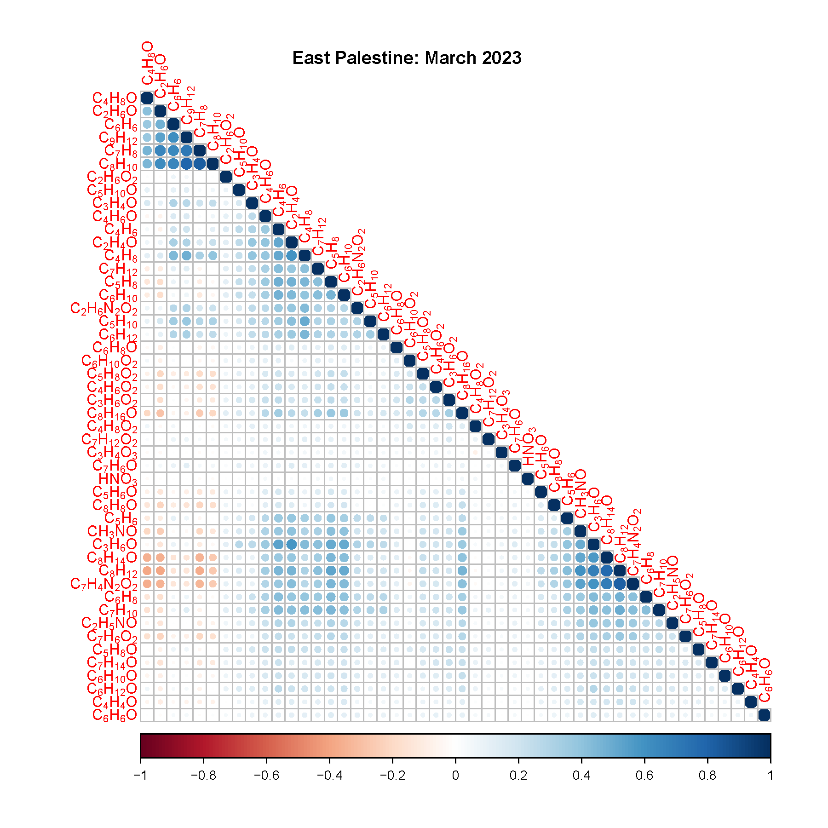
**

**
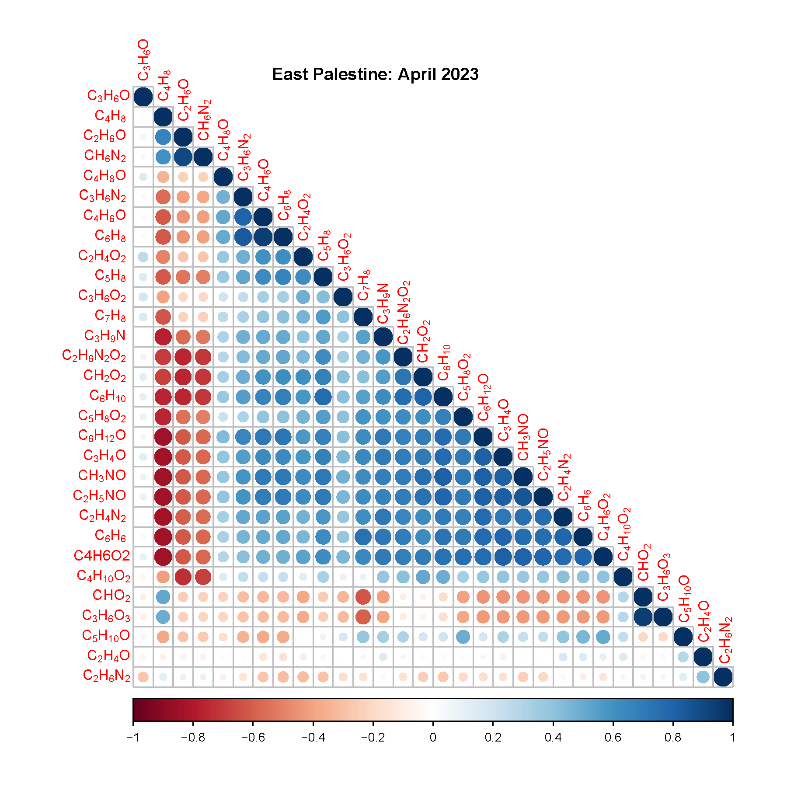
Figure S6.** East Palestine April 2023 correlation plot.


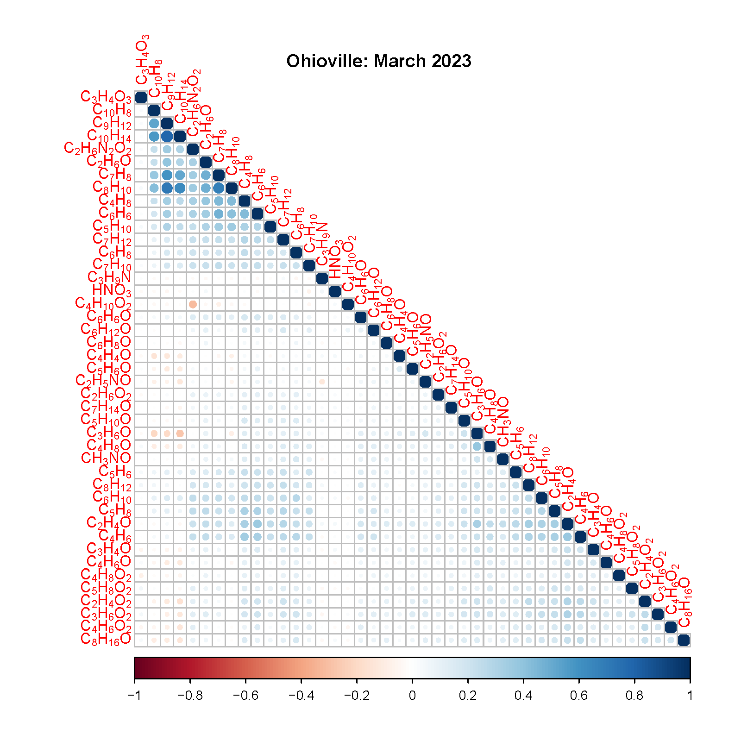
**Figure S7.** Ohioville March 2023 correlation plot.

**
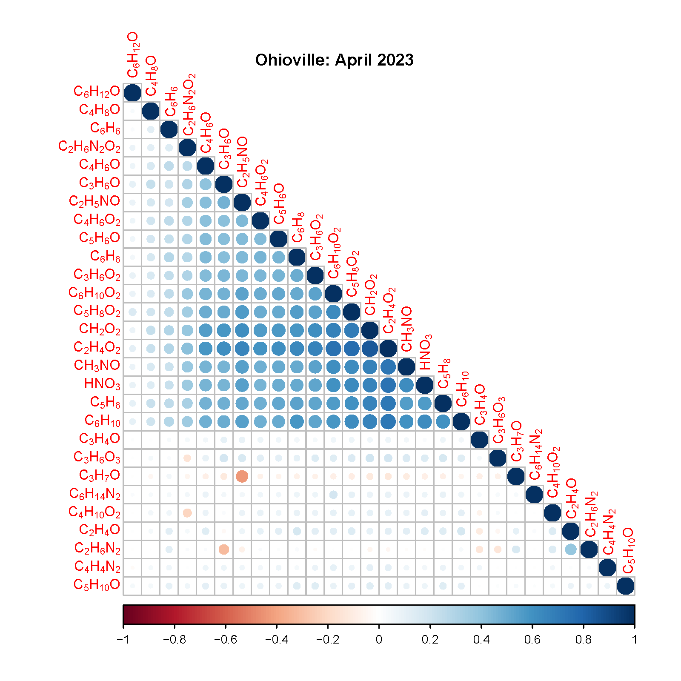
Figure S8.** Ohioville April 2023 correlation plot.

- 1. **Venn Diagram**

**Figure S9.** Venn diagram of unique and common VOCs for March and April 2023 campaigns.

**
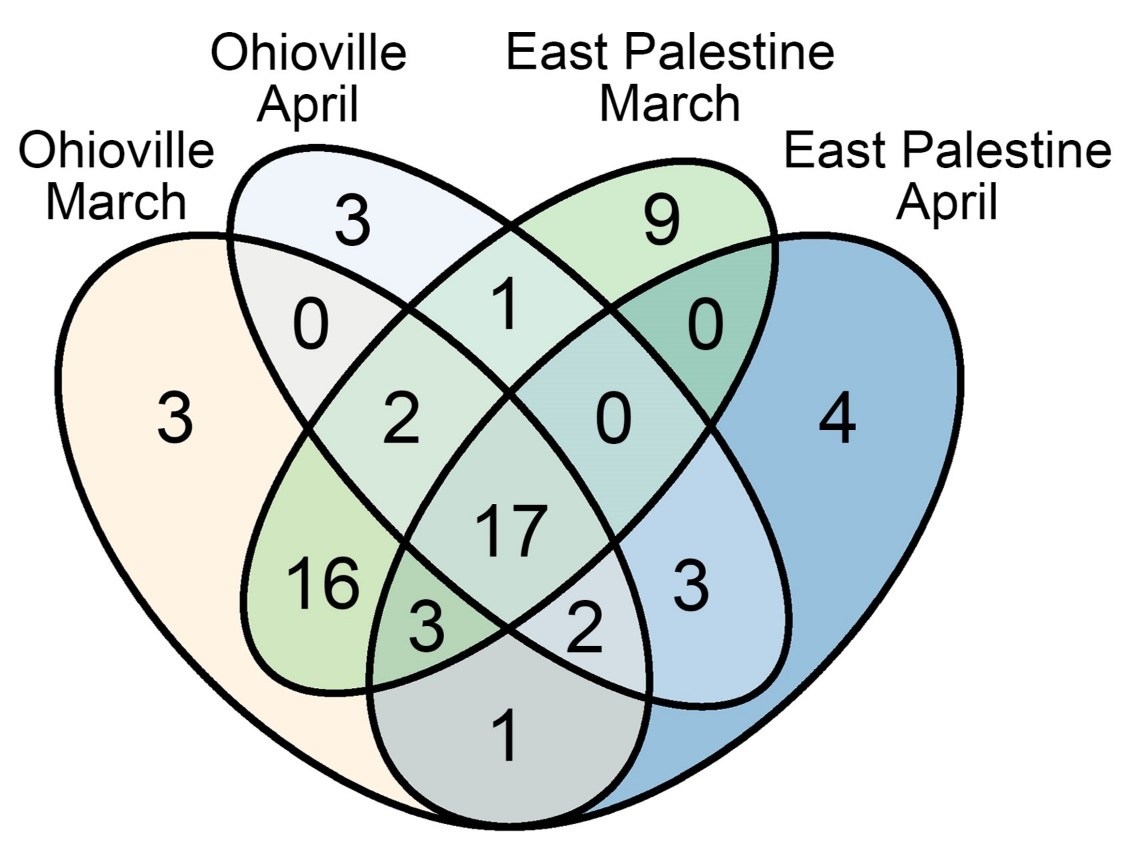
**

- 1. **Relative Concentration Route Maps for March and April 2023 Campaigns.**

**Figure S10.** Maps with East Palestine and Ohioville sampling routes for four VOCs


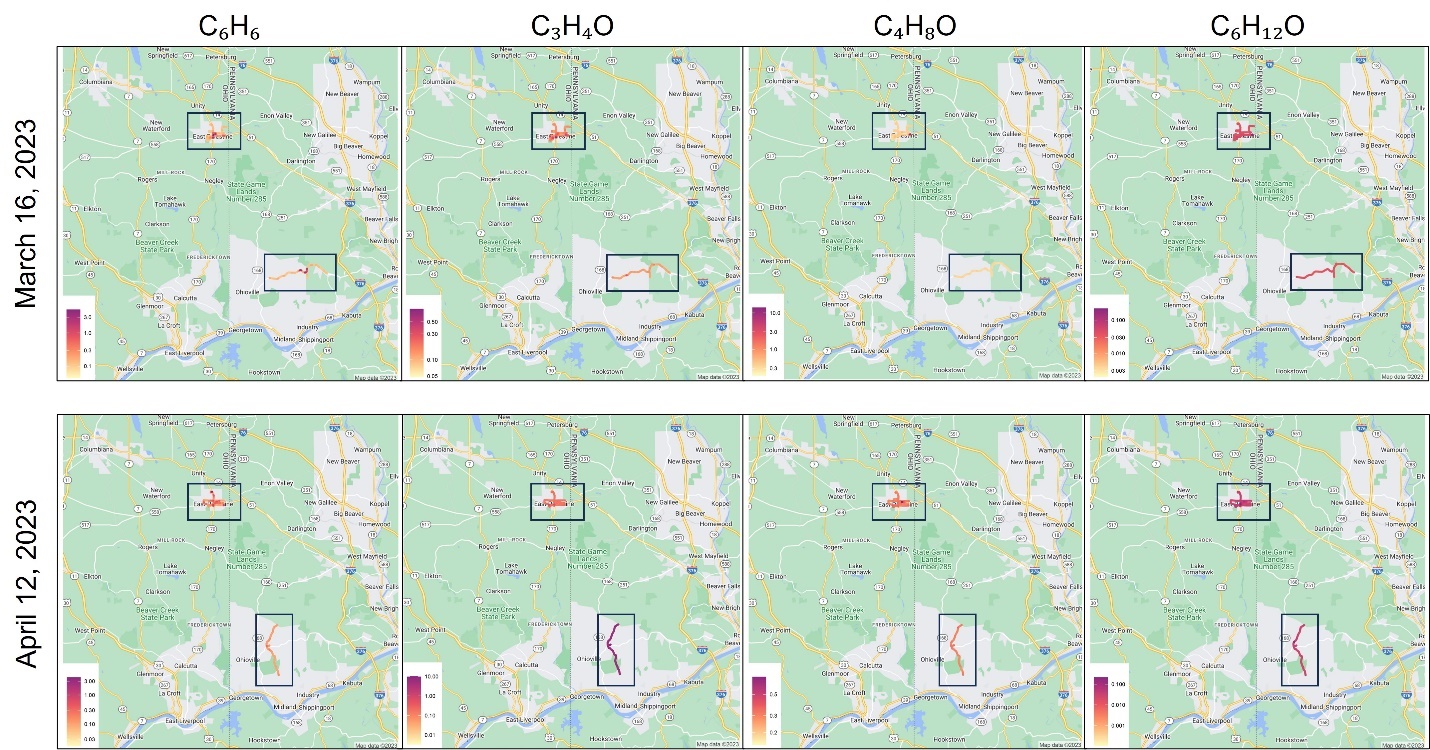


**Figure S11.** Maps with East Palestine and Ohioville sampling routes of February compounds that portrayed spatial patterns.


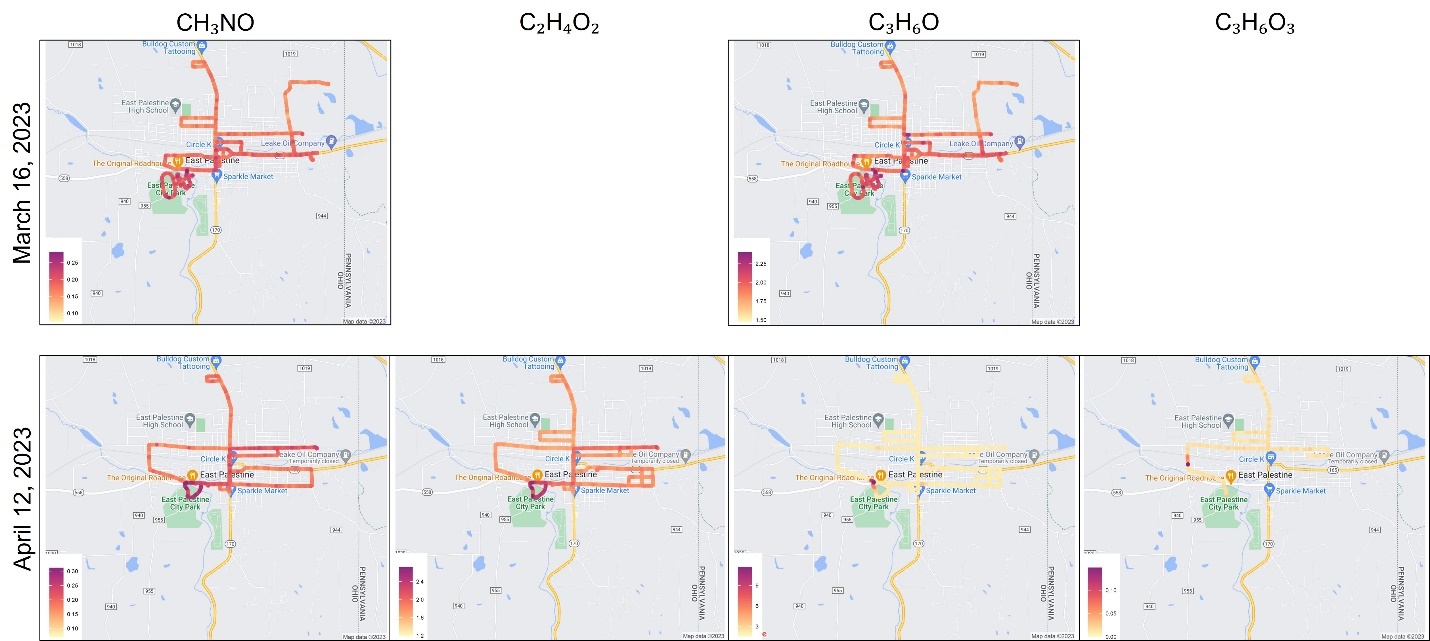


- 1. **Hazard Risk Charts for East Palestine**3

**Figure S12.** Hazard pie chart for March East Palestine.


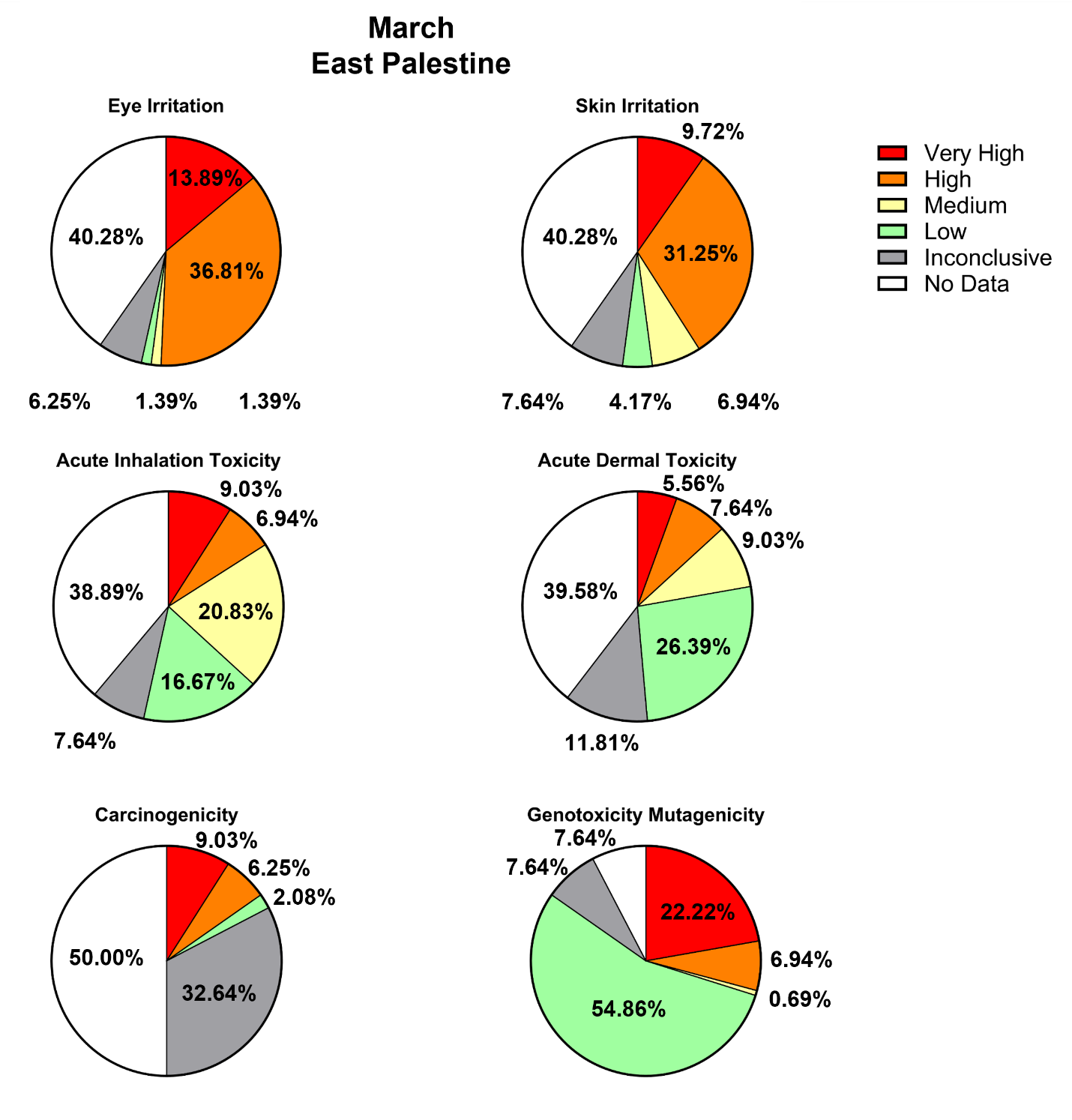


**Figure S13.** Hazard pie chart for April East Palestine.

**
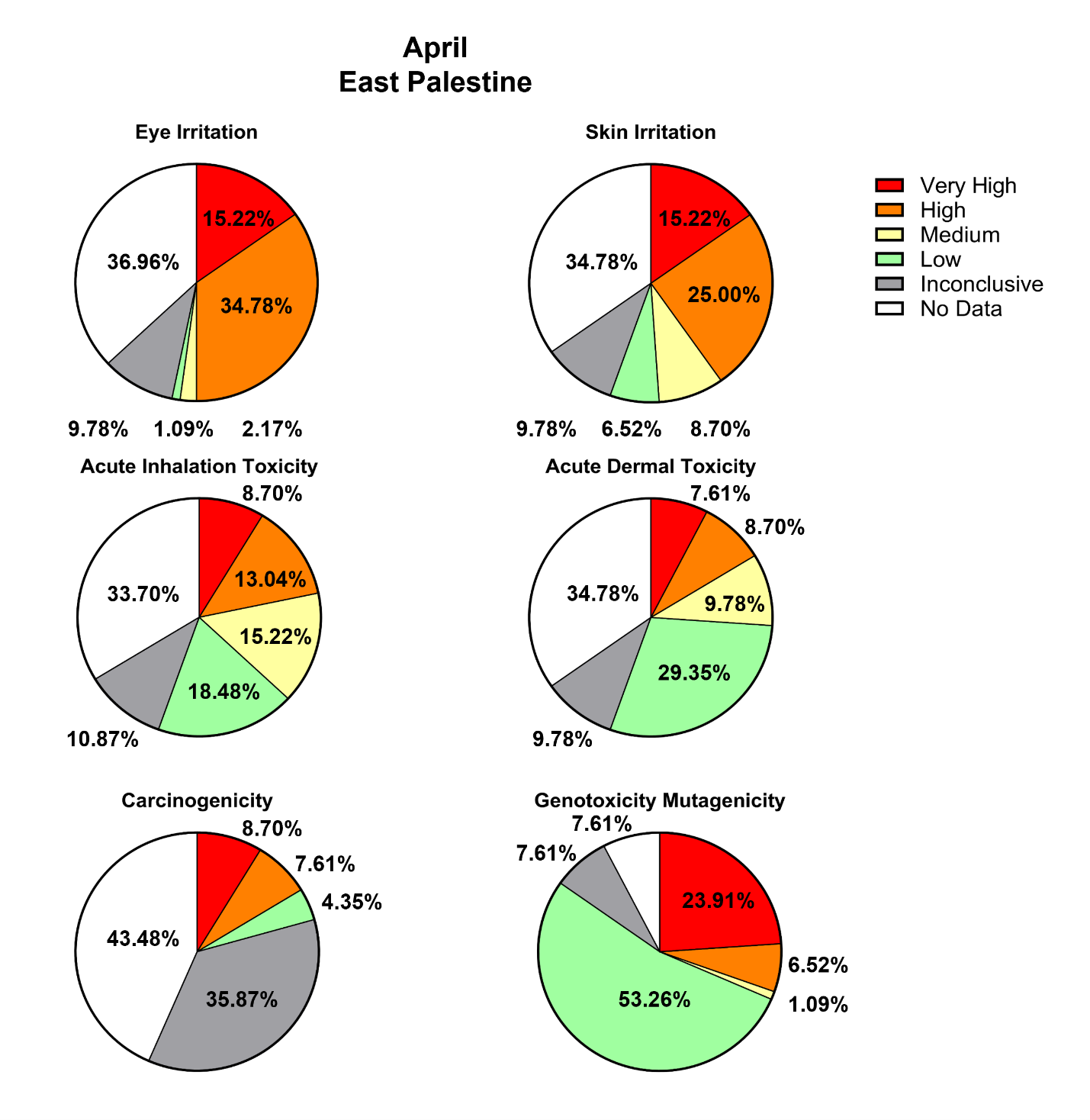
**

**5.5 Rscripts for Data Visualization**

**Script 1.** East Palestine and Ohioville script for visualizations for each town.

```{r setup, include=FALSE}

knitr::opts_chunk$set(echo = TRUE)

library(tidyverse)

library(ggmap)

library(readxl)

library(lubridate)

library(ggpubr)

library(googleway)

library(corrplot)

library(ggthemes)

# library(ggimage)

# library(sf)

# library(tigris)

googlemapskey <- "AIzaSyCLzwGw4ahSHLPcoy_tVwrUvRiQuXUV-wY"

register_google(key = googlemapskey)

latlonratio <- 364338.74/276725.00

site.location <- data.frame(lon= -80.5222838, lat=40.8360395,

image="fire_1f525.png")

eastpalestine <- "East Palestine, OH"

east.palestine.location <- geocode(eastpalestine)

eastpalestinemap <- qmap(eastpalestine,zoom=14,maptype="roadmap")

ohioville <- "Ohioville, PA"

ohioville.location <- geocode(ohioville)

ohiovillemap <- qmap(ohioville,zoom=14,maptype="roadmap")

midpoint.location<-apply(rbind(east.palestine.location,ohioville.location),2,mean)

midpointmap <- qmap(midpoint.location,zoom=11,maptype="roadmap")

lat.to.ft <- 364338.74

lon.to.ft <- 276725.00

lat.to.miles <- lat.to.ft / 5280

lon.to.miles <- lon.to.ft / 5280

binwidth.ft <- 150

binwidth.lat <- binwidth.ft/lat.to.ft

binwidth.lon <- binwidth.ft/lon.to.ft

```

## Load files

```{r list sheets in Excel file}

filename <- ".xlsx"

#~/East Palestine_Ohio_2023/_2023_Trip

file.tabs <- excel_sheets(filename)

print(file.tabs)

```

## East Palestine

### Data processing

```{r load dataset, message=FALSE}

loc.label <- "East Palestine"

voc.tabname <- file.tabs[]

met.tabname <- file.tabs[]

chem.tabname <- file.tabs[]

zoomlevel <- 14

cyclestocut <- 60*10 # 10 minutes

voc.data <- read_excel(filename,sheet=voc.tabname)

# Change timezone to US Central

voc.data$AbsTime <- as.POSIXct(as.character(voc.data$AbsTime),

tz="US/Central")

# Adjust timestamp so each row is unique

uniquetime <- !duplicated(voc.data$AbsTime)

delta <- round(mean(seconds(voc.data$AbsTime[uniquetime] -

(voc.data$AbsTime[1]+

voc.data$RelTime[uniquetime]))))

voc.data$DateTime <- round(voc.data$AbsTime[1]+

voc.data$RelTime + delta)

# Remove beginning artifacts

voc.data <- subset(voc.data,Cycle > cyclestocut)

# get rid of other time columns to avoid confusion

voc.data <- voc.data[,-(1:3)]

# Get compound names

cmp.names <- read_excel(filename,sheet=chem.tabname,col_names=FALSE)

names(cmp.names) <- c("detected mass","detected formula","chemical formula","chemical names")

chemformulas <- cmp.names$`chemical formula`

names(chemformulas) <- cmp.names$`detected mass`

# rename voc data columns

names(voc.data)[names(voc.data) %in% names(chemformulas)] <-

chemformulas[names(voc.data)[names(voc.data) %in% names(chemformulas)]]

# Met data

met.data <- read_excel(filename,sheet=met.tabname)

# Change timezone to US Central Daylight Time (file is in UTC)

met.data$DateTime <- with_tz(parse_date_time(met.data$TIME,

"%y-%m-%d%%H:%M:%S"),

tz="US/Central")

# round to nearest second

met.data$DateTime <- round(met.data$DateTime)

gps.mat <- str_split(met.data$GPSLOCATION,":",simplify = TRUE)

met.data$latitude <- as.numeric(gps.mat[,1])

met.data$longitude <- as.numeric(gps.mat[,2])

met.data$elevation <- as.numeric(gps.mat[,3])

# get distance from EP

met.data$x.EP.mi <- (met.data$longitude - east.palestine.location$lon)*lon.to.miles

met.data$y.EP.mi <- (met.data$latitude - east.palestine.location$lat)*lat.to.miles

met.data$dist.EP.mi <- sqrt(met.data$x.EP.mi^2+met.data$y.EP.mi^2)

# Combine VOC and GPS data

voc.data <- left_join(voc.data,met.data[,c("DateTime","longitude","latitude","x.EP.mi","y.EP.mi","dist.EP.mi")])

voc.data$location <- loc.label

# Make into "long" data frame

voc.data.df <- pivot_longer(voc.data,cols=

all_of(as.character(chemformulas)))

write.csv(voc.data,paste0(voc.tabname,".data.csv"),row.names = FALSE)

write.csv(voc.data.df,paste0(voc.tabname,".data.longformat.csv"),row.names = FALSE)

#Merging segmented East Palestine VOC Files (Added when undergoing April 2023 data analysis)

EPMedia <- read.csv("EP_Media_TIII_041223.data.longformat.csv")

EP_Pt1<-read.csv("EP_Pt_1_TIII_041223.data.longformat.csv")

EP_Pt2<- read.csv("EP_Pt_2_TIII_041223.data.longformat.csv")

EP_Pt4<- read.csv("EP_Pt_4_TIII_041223.data.longformat.csv")

media_pt1<-rbind(EPMedia,EP_Pt1)

media_pt1_pt2<-rbind(media_pt1,EP_Pt2)

voc.data.df<-rbind(media_pt1_pt2,EP_Pt4)

#re-save in longformat

write.csv(voc.data.df, paste0("East_Palestine_TII_or_TIII_.data.longformat.csv"),row.names = FALSE)

#convert to wide format (for voc.data)

voc.data.df<- read.csv("East_Palestine_TII_or_TIII_.data.longformat.csv")

voc.data<-pivot_wider(voc.data.df)

#replace NA's to 0 in wide format file (voc.data)

#voc.data.final<-replace(voc.data,is.na(voc.data),0)

#write.csv(voc.data.final,paste0("East_Palestine_TII_or_TIII_.data.csv"),row.names = FALSE)

#for the rest of the code

voc.data<-read.csv("East_Palestine_TII_or_TIII_.data.csv")

voc.data.df<-read.csv("East_Palestine_TII_or_TIII_.data.longformat.csv")

#Merging Compound Names

# Get compound names

library("readxl")

cmp.names <- read_excel("East_Palestine_TII_or_TIII_Cmpd_Names.xlsx",col_names=FALSE)

names(cmp.names) <- c("detected formula","chemical formula","chemical names")

chemformulas <- cmp.names$`chemical formula`

#names(chemformulas) <- cmp.names$`detected mass`

```

### Basic summary plots across chemicals

```{r basic summary plots, message=FALSE}

# locations

location <- apply(voc.data[,c("longitude","latitude")],2,median)

locmap <- qmap(location,zoom=zoomlevel,maptype="roadmap")

drivemap <- locmap +

geom_point(aes(x=longitude,y=latitude,color=DateTime),data=voc.data)+

ggtitle("East_Palestine_041223")+

theme(legend.position = "none")

ggsave(paste0("East_Palestine_TII_or_TIII_041223.locations.pdf"),drivemap)

# Correlation matrix Spearman = cor(method = c("spearman")))

voc.corr <- voc.data %>% select(all_of(as.character(chemformulas))) %>% cor(method=c("spearman"))

pdf(paste0("East_Palestine_TIII_041223_Spearman_NA.corr.pdf"),height=9,width=9)

voc.corr.plt <- corrplot(voc.corr,method = 'circle', type = 'lower',

order = 'hclust', diag=TRUE,title= "East_Palestine_TIII_041223")

dev.off()

chemorder <- rownames(voc.corr.plt$corr)

# Re-order based on clustering

voc.data.df$name <- factor(voc.data.df$name,

levels=chemorder)

# timecourse

timecourse <- ggplot(voc.data.df)+

geom_point(aes(x=DateTime,y=value),size=0.5)+

facet_wrap(~name,scales="free_y")+ggtitle("East_Palestine_TIII_041223")

ggsave(paste0("East_Palestine_TII_or_TIII_041223.timecourse.pdf"),timecourse,

height=10,width=12)

boxplots <- ggplot(voc.data.df)+

geom_boxplot(aes(x=value,y=name),outlier.size = 0.1)+

scale_x_log10()+annotation_logticks(side="b")+ggtitle("East_Palestine_TII_or_TIII_041223")

ggsave(paste0("East_Palestine_TIII_041223.boxplots.pdf"),boxplots,

height=10,width=8)

```

### Raw maps for each chemical

```{r raw maps, message=FALSE,warning=FALSE}

pdf(paste0("East_Palestine_TII_or_TIII_041223.rawmaps.pdf"),height=8,width=6)

for (chem in chemorder) {

data.tmp <- subset(voc.data.df,name==chem)

data.tmp <- data.tmp[order(data.tmp$value),] # Put higher values on top

pmap <- locmap +

geom_point(aes(x=longitude,y=latitude,color=value),

data=data.tmp)+

scale_color_viridis_c("",option="magma",direction=-1,begin=0.4)+

theme_map()+

ggtitle(paste(chem,"East_Palestine_TII_or_TIII_041223"))

ptime <- ggplot(data.tmp) +

geom_point(aes(x=DateTime,y=value,color=value))+

scale_color_viridis_c("",option="magma",direction=-1,begin=0.4)+

theme(legend.position = "none")

pbox <- ggplot(data.tmp)+geom_boxplot(aes(y=value,x=DateTime))+ylab("")+

xlab("")+scale_x_datetime(breaks=median(data.tmp$DateTime),labels="")

pall <- ggarrange(pmap,

ggarrange(ptime,pbox,widths=c(4,1),nrow=1),

heights=c(4,1),nrow=2)

print(pall)

}

dev.off()

```

## Ohioville

```{r load dataset, message=FALSE}

loc.label <- "Ohioville"

voc.tabname <- file.tabs[1]

met.tabname <- file.tabs[2]

chem.tabname <- file.tabs[13]

zoomlevel <- 13

cyclestocut <- 60*10 # 10 minutes

voc.data <- read_excel(filename,sheet=voc.tabname)

# Change timezone to US Central Daylight Time

voc.data$AbsTime <- as.POSIXct(as.character(voc.data$AbsTime),

tz="US/Central")

# Adjust timestamp so each row is unique

uniquetime <- !duplicated(voc.data$AbsTime)

delta <- round(mean(seconds(voc.data$AbsTime[uniquetime] -

(voc.data$AbsTime[1]+

voc.data$RelTime[uniquetime]))))

voc.data$DateTime <- round(voc.data$AbsTime[1]+

voc.data$RelTime + delta)

# Remove beginning artifacts

voc.data <- subset(voc.data,Cycle > cyclestocut)

# get rid of other time columns to avoid confusion

voc.data <- voc.data[,-(1:3)]

# Get compound names

cmp.names <- read_excel(filename,sheet=chem.tabname,col_names=FALSE)

names(cmp.names) <- c("detected mass","detected formula","chemical formula","chemical names")

chemformulas <- cmp.names$`chemical formula`

names(chemformulas) <- cmp.names$`detected mass`

# rename voc data columns

names(voc.data)[names(voc.data) %in% names(chemformulas)] <-

chemformulas[names(voc.data)[names(voc.data) %in% names(chemformulas)]]

# Met data

met.data <- read_excel(filename,sheet=met.tabname)

# Change timezone to US Central Daylight Time (file is in UTC)

met.data$DateTime <- with_tz(parse_date_time(met.data$TIME,

"%y-%m-%d%%H:%M:%S"),

tz="US/Central")

# round to nearest second

met.data$DateTime <- round(met.data$DateTime)

gps.mat <- str_split(met.data$GPSLOCATION,":",simplify = TRUE)

met.data$latitude <- as.numeric(gps.mat[,1])

met.data$longitude <- as.numeric(gps.mat[,2])

met.data$elevation <- as.numeric(gps.mat[,3])

# get distance from EP

met.data$x.EP.mi <- (met.data$longitude - east.palestine.location$lon)*lon.to.miles

met.data$y.EP.mi <- (met.data$latitude - east.palestine.location$lat)*lat.to.miles

met.data$dist.EP.mi <- sqrt(met.data$x.EP.mi^2+met.data$y.EP.mi^2)

# Combine VOC and GPS data

voc.data <- left_join(voc.data,met.data[,c("DateTime","longitude","latitude","x.EP.mi","y.EP.mi","dist.EP.mi")])

voc.data$location <- loc.label

# Make into "long" data frame

voc.data.df <- pivot_longer(voc.data,cols=

all_of(as.character(chemformulas)))

write.csv(voc.data,paste0(voc.tabname,".data.csv"),row.names = FALSE)

write.csv(voc.data.df,paste0(voc.tabname,".data.longformat.csv"),row.names = FALSE)

```

### Basic summary plots across chemicals

```{r basic summary plots, message=FALSE}

# locations

location <- apply(voc.data[,c("longitude","latitude")],2,median)

locmap <- qmap(location,zoom=zoomlevel,maptype="roadmap")

drivemap <- locmap +

geom_point(aes(x=longitude,y=latitude,color=DateTime),data=voc.data)+

ggtitle(voc.tabname)

ggsave(paste0(voc.tabname,".locations.pdf"),drivemap)

# Correlation matrix (make two plots: 1 with pearson cor() & 1 with spearman cor(method = c("spearman")))

voc.corr <- voc.data %>% select(all_of(as.character(chemformulas))) %>% cor()

pdf(paste0(voc.tabname,".corr.pdf"),height=8,width=8)

voc.corr.plt <- corrplot(voc.corr,method = 'circle', type = 'lower',

order = 'hclust', diag=TRUE,title=voc.tabname)

dev.off()

chemorder <- rownames(voc.corr.plt$corr)

# Re-order based on clustering

voc.data.df$name <- factor(voc.data.df$name,

levels=chemorder)

# timecourse

timecourse <- ggplot(voc.data.df)+

geom_point(aes(x=DateTime,y=value),size=0.5)+

facet_wrap(~name,scales="free_y")+ggtitle(voc.tabname)

ggsave(paste0(voc.tabname,".timecourse.pdf"),timecourse,

height=8,width=10)

boxplots <- ggplot(voc.data.df)+

geom_boxplot(aes(x=value,y=name),outlier.size = 0.1)+

scale_x_log10()+annotation_logticks(side="b")+ggtitle(voc.tabname)

ggsave(paste0(voc.tabname,".boxplots.pdf"),boxplots,

height=10,width=8)

```

### Raw maps for each chemical

```{r raw maps, message=FALSE, warning=FALSE}

pdf(paste0(voc.tabname,".rawmaps.pdf"),height=8,width=6)

for (chem in chemorder) {

data.tmp <- subset(voc.data.df,name==chem)

data.tmp <- data.tmp[order(data.tmp$value),] # Put higher values on top

pmap <- locmap +

geom_point(aes(x=longitude,y=latitude,color=value),

data=data.tmp)+

scale_color_viridis_c("",option="magma",direction=-1,begin=0.4)+

theme_map()+

ggtitle(paste(chem,voc.tabname))

ptime <- ggplot(data.tmp) +

geom_point(aes(x=DateTime,y=value,color=value))+

scale_color_viridis_c("",option="magma",direction=-1,begin=0.4)+

theme(legend.position = "none")

pbox <- ggplot(data.tmp)+geom_boxplot(aes(y=value,x=DateTime))+ylab("")+

xlab("")+scale_x_datetime(breaks=median(data.tmp$DateTime),labels="")

pall <- ggarrange(pmap,

ggarrange(ptime,pbox,widths=c(4,1),nrow=1),

heights=c(4,1),nrow=2)

print(pall)

}

dev.off()

```

**Script 2.** East Palestine and Ohioville Comparison maps.

```{r setup, include=FALSE}

knitr::opts_chunk$set(echo = TRUE)

library(tidyverse)

library(ggmap)

library(readxl)

library(lubridate)

library(ggpubr)

library(googleway)

library(corrplot)

library(ggthemes)

library(scales)

# library(ggimage)

# library(sf)

# library(tigris)

googlemapskey <- "AIzaSyCLzwGw4ahSHLPcoy_tVwrUvRiQuXUV-wY"

register_google(key = googlemapskey)

latlonratio <- 364338.74/276725.00

site.location <- data.frame(lon= -80.5222838, lat=40.8360395,

image="fire_1f525.png")

eastpalestine <- "East Palestine, OH"

east.palestine.location <- geocode(eastpalestine)

eastpalestinemap <- qmap(eastpalestine,zoom=14,maptype="roadmap")

ohioville <- "Ohioville, PA"

ohioville.location <- geocode(ohioville)

ohiovillemap <- qmap(ohioville,zoom=14,maptype="roadmap")

midpoint.location<-apply(rbind(east.palestine.location,ohioville.location),2,mean)

midpointmap <- qmap(midpoint.location,zoom=11,maptype="roadmap")

lat.to.ft <- 364338.74

lon.to.ft <- 276725.00

lat.to.miles <- lat.to.ft / 5280

lon.to.miles <- lon.to.ft / 5280

binwidth.ft <- 150

binwidth.lat <- binwidth.ft/lat.to.ft

binwidth.lon <- binwidth.ft/lon.to.ft

```

## Load files

```{r list sheets in Excel file}

filename <- ".xlsx"

file.tabs <- excel_sheets(filename)

print(file.tabs)

```

```{r get and merge data from two locations}

voc.data.df <- rbind(

read.csv(paste0(file.tabs[1],".data.longformat.csv")),

read.csv("East_Palestine_TII_or_TIII.data.longformat.csv"))

voc.data.df$DateTime <- as.POSIXct(as.character(voc.data.df$DateTime),

tz="US/Central")

chems <- unique(voc.data.df$name)

```

## Maps and boxplots comparing the two

```{r raw maps, message=FALSE, warning=FALSE}

pdf(paste0("comparison.rawmaps.pdf"),height=8,width=6)

for (chem in chems) {

data.tmp <- subset(voc.data.df,name==chem)

data.tmp <- data.tmp[order(data.tmp$value),] # Put higher values on top

pmap <- midpointmap +

geom_point(aes(x=longitude,y=latitude,color=value),

data=data.tmp,size=0.5)+

scale_color_viridis_c("",option="magma",direction=-1,begin=0.4,trans=log10_trans())+

theme_map()+

ggtitle(paste(chem))

ptime <- ggplot(data.tmp) +

geom_point(aes(x=DateTime,y=value,color=value))+

scale_color_viridis_c("",option="magma",direction=-1,begin=0.4,trans=log10_trans())+scale_y_log10()+

theme(legend.position = "none",

strip.text.x = element_text(size = 8))+

facet_wrap(~location,scales="free_x",nrow=1)

pbox <- ggplot(data.tmp)+geom_boxplot(aes(y=value,x=DateTime))+

xlab("")+scale_x_datetime(breaks=median(data.tmp$DateTime),labels="")+

scale_y_log10()+

theme(axis.text = element_text(color="white"),

axis.title.y = element_blank(),

axis.text.y = element_blank(),

strip.text.x = element_text(size = 8))+

facet_wrap(~location,scales="free_x",nrow=1)

pall <- ggarrange(pmap,

ggarrange(ptime,pbox,widths=c(2,1),nrow=1),

heights=c(4,1),nrow=2)

print(pall)

}

dev.off()

```

1. **Magellan MX500 Weather Figures**

The Magellan MX500 collects GPS coordinates in addition to wind speed (mph), wind direction (in degrees), temperature (°F) and relative humidity (%). These are the four types of meteorological data considered and visualized during mobile air sampling campaigns.

- 1. **Temperature and Relative Humidity Comparisons**

**
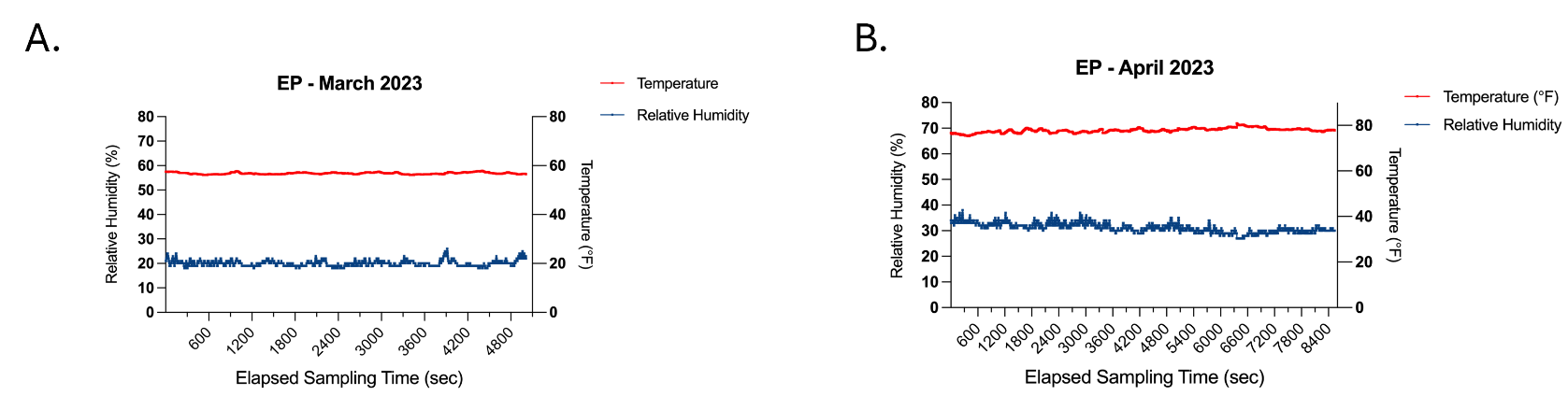
Figure S14.** Relative humidity and temperature measurements in real-time during sampling acquisition in A) March and B) April.

- 1. **Temperature, Relative Humidity, Wind Speed and Direction Comparisons**

**Figure S15.** Comparison of weather parameters across sampling months in East Palestine. Comparison of A) relative humidity, B) temperature, C) wind speed, and D-E) wind direction between March and April sampling campaigns. Average wind direction is indicated by the line extending from the circular plot. **** p<0.0001.


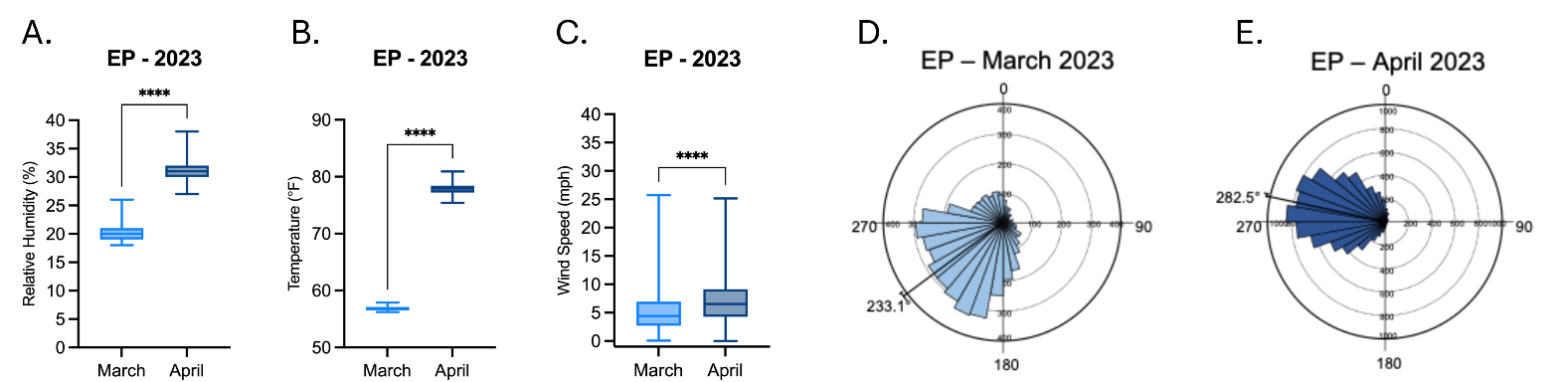


- 1. **Spearman Correlations for Relative Humidity and Temperature to VOCs**

**Figure S16.** Spearman’s correlation matrix of detected VOCs with weather parameters. Correlation matrix displaying only significantly (p<0.01) correlated relationships in A) March and B) April between the detected VOCs, relative humidity, and temperature.

**
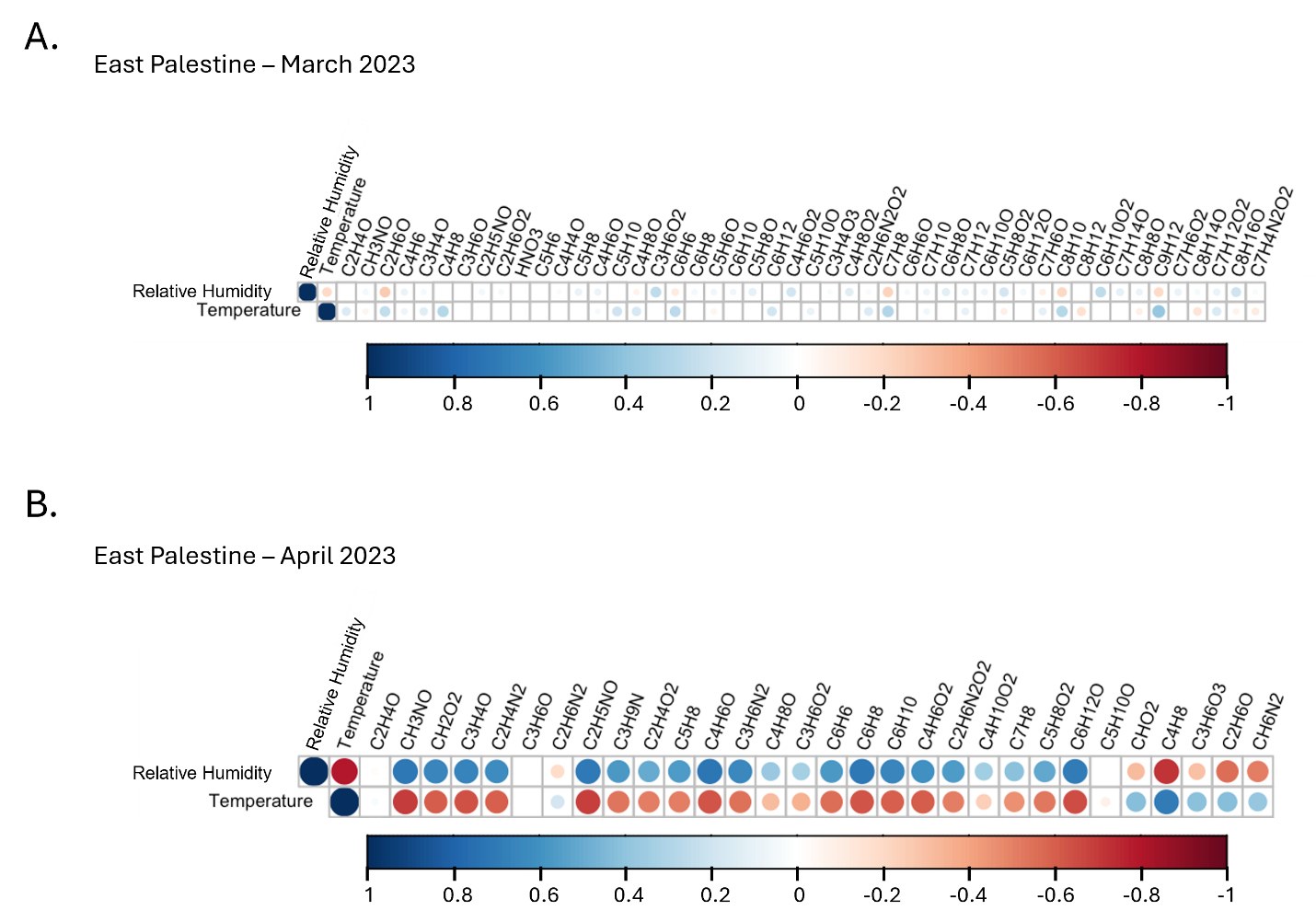
**

**References**

1. Kansas State University. Center for Hazardous Substance Research: Understanding Units of Measurement. Kansas State University **2006**.chrome-extension://efaidnbmnnnibpcajpcglclefindmkaj/https://cfpub.epa.gov/ncer_abstracts/index.cfm/fuseaction/display.files/fileid/14285
2. Oladeji, O.; Saitas, M.; Mustapha, T.; Tasoglou, A.; Johnson, N.M.; Chiu, W.A.; Rusyn, I.; Robinson, A.L.; Presto, A.A. Supplemental Information: Air Pollutant Patterns and Human Health Risk Following the East Palestine, Ohio Train Derailment*. Environmental Science & Technology Letters* **2023**, *10* (8), 680-685. https://doi.org/10.1021/acs.estlett.3c00324.
3. Environmental Protection Agency. The Cheminformatics Module **2023**. https://www.epa.gov/comptox-tools/cheminformatics
